# Electroacupuncture as a rapid-onset and safer complementary therapy for depression: A systematic review and meta-analysis

**DOI:** 10.3389/fpsyt.2022.1012606

**Published:** 2023-01-06

**Authors:** Zhinan Zhang, Xiaowen Cai, Yuying Liang, Rui Zhang, Xinyu Liu, Liming Lu, Yong Huang

**Affiliations:** ^1^School of Traditional Chinese Medicine, Southern Medical University, Guangzhou, Guangdong, China; ^2^Medical College of Acu-Moxi and Rehabilitation, Guangzhou University of Chinese Medicine, Guangzhou, Guangdong, China

**Keywords:** rapid-onset, safety, electroacupuncture, depression, meta-analysis

## Abstract

**Background:**

Electroacupuncture (EA) is a promising therapy for depression. However, a comprehensive review of EA for depression is needed.

**Methods:**

We conducted a systematic review and meta-analysis in accordance with the Preferred Reporting Items for Systematic Reviews and Meta-analyses (PRISMA 2020) guidelines to evaluate the efficacy and safety of EA for depression. Potentially relevant trials and reviews were searched in MEDLINE, EMBASE, PsycINFO, and CENTRAL from inception to March 2022. EA alone and combined with other therapy were eligible for inclusion. The severity of depression during and after treatment and the number of adverse events were assessed as outcomes. Risk of bias (ROB) evaluation, subgroup analysis, sensitivity analysis, reporting bias assessment, and GRADE system evaluation were also conducted.

**Results:**

Thirty-four trials were included. The overall ROB was medium. Low-quality evidence showed that the efficacy of EA was not less than that of antidepressants [EA + selective serotonin reuptake inhibitors (SSRIs) and tricyclic antidepressants (TCAs)] and manual acupuncture (MA). EA and EA + SSRIs had better efficacy than SSRIs alone in decreasing the severity of depression during the early treatment. Moderate-quality evidence also showed that EA and EA + SSRIs were safer than SSRIs alone. Sensitivity analysis was mostly not feasible. Major publication bias was unlikely.

**Conclusion:**

These results indicate that the efficacy of EA is not less than that of antidepressants and MA. Moreover, EA and EA + SSRI treatments show a more rapid onset and greater safety than SSRIs. More high-quality trials are needed for further confirmation.

**Systematic review registration:**

[www.crd.york.ac.uk/prospero/display_ record.php?RecordID=329143], identifier [CRD42022329143].

## 1. Introduction

Depression, an emerging concern in modern communities (1), is a mood disorder characterized by a constantly low mood and may be accompanied by sadness or irritability, sleeping disorders, appetite loss, libido disorders, constipation, and low self-esteem (2). More importantly, depression is the leading cause of suicide and has been linked to disability and other disease burdens (3).

The mechanisms underlying the therapeutic effects of EA are still not fully understood. Efforts have been made to explore these mechanisms (4), and possible mechanisms include reduction of inflammation (5), regulation of endocrine activity (6), increasing neurotransmitters (7), and increasing synaptic plasticity (8). Although the mechanism of action of EA is not clear, a multitarget mode of activity seems to be a reasonable explanation. Nevertheless, mechanism studies can provide fundamental research evidence for the efficacy of EA in depression.

The first-line medicines for depression include SNRIs, selective NARIs, and SSRIs, which block the reuptake of neurotransmitters and increase their concentration in the synaptic cleft (9). SSRIs are the most commonly used medications for depression (10). However, these medications have some concerning limitations. About 1/3rd of the patients do not respond to medications (11). Moreover, the onset of their therapeutic effects may require more than 1 week (12). Therefore, alternative rapid-acting therapeutic agents are required for clinical practice (13).

Electroacupuncture (EA) involves the passage of a small electric current between pairs of acupuncture needles (14). For years, more and more RCTs examined the effectiveness and safety of EA in depression, which provided some evidence indicating that EA may serve as an alternative for antidepressants. However, the efficacy and safety of EA in depression remains debatable. A review in 2018 exploring the overall efficacy of acupuncture (both EA and MA) on depression found weak evidence suggesting that the efficacy and safety of acupuncture was no less than that of antidepressants, and that acupuncture combined with antidepressants may have better efficacy than antidepressants alone (15). Some reviews focusing on EA for specific subtype of depression, such as PSD (16) postpartum depression (17, 18) found EA had similar efficacy to other therapy and better safety. But most of these reviews did not carry subgroups comparison and the evidence grade was low mainly due to lack of included studies. On the other hand, these reviews are outdated. After their publication, several RCTs data came up providing newer and higher-quality evidence for systematic review and meta-analysis. We also found week evidence indicating that EA had rapid-onset efficacy for depression in 2016 (19, 20). Considering the possible rapid-onset efficacy of EA, we notice that it lacks of review updating the comparison of the efficacy of EA and other therapies in different time point, such as early period of the treatment. Therefore, a comprehensive review exploring the efficacy of EA on depression in different phase of treatment with latest RCTs updated data is required to support the effectiveness and safety of EA for depression.

Considering the increasing need for alternative therapeutic modalities for depression, and possible rapid-onset efficacy of EA, it may be a promising method for treating depression. Some RCTs have been published since the latest comprehensive review in 2018 (15). Moreover, the last reviews only included 14 eligible trials and more RCTs are needed to improve the evidence level and focused on subtype of depression. Therefore, in this review, we planned to conduct a meta-analysis to evaluate and update the efficacy of EA for depression in different time points and safety by including data from the latest trials to explore the rapid-onset efficacy and safety of EA as complementary therapy for depression.

## 2. Materials and methods

The study was conducted in accordance with the Preferred Reporting Items for Systematic Reviews and Meta-analyses (PRISMA 2020) guidelines (21). This review has been registered on PROSPERO (CRD42022329143^1^).

### 2.1. Criteria for considering studies for this review

#### 2.1.1. Types of studies

We sought to include parallel-group randomized trials, irrespective of their publication status and language of publication. Cluster-randomized trials, controlled before-and-after studies, case reports, cohort studies, and other non-randomized studies were excluded.

#### 2.1.2. Types of participants

We included trials with and without sex and age restrictions. Patients should have been diagnosed with depression or developed clinical symptoms of depression using the following criteria: the DSM; the RDC; the International Statistical Classification of Diseases and Related Health Problems (ICD); the CCMD; If necessary, the definitions of trial authors. Trials on patients with comorbidities were also included as long as depression was the main focus of the study. All treatment settings were eligible for inclusion.

#### 2.1.3. Types of interventions

##### 2.1.3.1. Experimental interventions

Studies evaluating EA and combinations of EA with other eligible therapies (e.g., EA + antidepressants, EA + psychological therapies) were eligible for inclusion.

##### 2.1.3.2. Comparator interventions

The eligible comparator interventions for inclusion were waitlist control; treatment as usual; placebo; sham acupuncture; MA; antidepressant medication; psychological therapies (cognitive-behavioral therapy, behavioral therapy, and psychotherapy, counseling); physical therapies; and combinations of two or more eligible therapies.

#### 2.1.4. Types of outcome measures

Depression severity scores rated as continuous data were eligible. When the trial adopted two or more scales, the hierarchy of outcome measures was as follows:

(1) Hamilton Depression Rating Scale (HAMD) (17- or 24-item versions) (22).
(2) Beck Depression Inventory (BDI) (23).
(3) Self-rating depression scale (SDS) (24).
(4) World Health Organization Quality of Life (WHOQOL) depression score (25).
(5) Short Form Health Survey (SF-36) depression score (26).
(6) Depression scores of other quality of life measures.
(7) Other depression scales.

The total number of adverse events was also eligible as an outcome to assess the safety of the interventions.

In our preliminary searching, we found that 6-weeks-treatment with measurement of every 2 weeks is the mostly-used paradigm. Therefore, weeks 2, 4, and 6 were chose to represent early, middle, and later phase time points.

The time points for outcome measurement were as follows:

(1) At the end of the treatment (right after the last time of treatment).
(2) At the end of week 2 during treatment.
(3) At the end of week 4 during treatment.
(4) At the end of week 6 during treatment.
(5) At the end of follow-up (If a study provided several follow-ups, we adopt the last data).

### 2.2. Search methods for identification of studies

MEDLINE, EMBASE, and PsycINFO were searched using the OVID database, and CENTRAL was searched using the Cochrane Library. To identify potentially relevant articles, we searched the databases for articles published from database inception to March 2022 with no restrictions on language. In brief, the key search terms were (electroacupuncture*) and (depression* or depressive disorder* or depressive disorder, major*). The search themes were adapted to each electronic database (Supplementary material). Relevant references of reviewed articles and the included studies were also searched.

### 2.3. Data collection and analysis

#### 2.3.1. Selection of studies

All of the retrieved trial records were reviewed by two authors (YL and XL) independently, with an extra author (LL) serving as a referee for differences in assessments by the two authors. After the duplicated records were removed, the two authors screened the title and abstract of the remaining article records to determine their eligibility. The full text of the remaining article records was reviewed and read for inclusion. We also recorded this process in the study flow diagram.

#### 2.3.2. Data extraction and management

After the trials were included, trial characteristics (methods, participants, interventions, and outcomes) and data (depression severity) were extracted and recorded by two authors (YL and XL). Besides, we contacted corresponding authors to ask for additional trial data if the published data were inadequate. We planned to conduct the following comparisons:

(1) EA vs. antidepressants.
(2) EA + antidepressants vs. antidepressants.
(3) EA vs. MA.
(4) EA + antidepressants vs. MA + antidepressants.
(5) EA vs. psychological therapies.
(6) EA + psychological therapies vs. psychological therapies.
(7) EA vs. physical therapies.
(8) EA + physical therapies vs. physical therapies.
(9) EA vs. control (waitlist control; treatment as usual; no treatment; placebo; sham acupuncture).

### 2.4. Assessment of the risk of bias in the included studies

Using the ROB 2.0 tools (new ROB evaluate method with reducing subjectivity bias of the reviewer by using standardized system and algorithm to generated ROB grades) (27), two review authors (YL and XL) independently assessed the ROB of every included trial in accordance with the criteria outlined in the Cochrane Handbook for Systematic Reviews of Interventions (28). An extra author (LL) served as a referee to address any differences in the assessments by the two reviewers. The ROB 2.0 assessment included the following six domains, and each domain was rated as low risk, some concerns, or high risk:

(1) Randomization process.
(2) Deviations from intended interventions.
(3) Missing outcome data.
(4) Measurement of outcomes.
(5) Selection of the reported result.
(6) Overall.

### 2.5. Measures of treatment effect

The data were pooled using Review Manager software (version 5.4). Continuous data were presented in terms of mean difference (MD) with 95% CIs and dichotomous data in terms of RR with 95% CIs. We also pooled the data obtained using different methods (e.g., HAMD and SDS) to measure the severity of depression due to their conceptual similarity.

### 2.6. Unit of analysis issues

When trials contained more than two EA groups (e.g., EA at acupoint A vs. EA at acupoint B vs. no treatment control), we combined data from both groups.

### 2.7. Dealing with missing data

When we encountered trials for which only the abstract was available, the data were presented as different values at different time points without other data to calculate the original mean difference. We also contacted the corresponding authors of the trials through email for more information on the trial characteristics and data details.

### 2.8. Assessment of heterogeneity

Heterogeneity was assessed using the *I*^2^ statistic and *P*-values through chi-square test. When *P* < 0.05, the criteria for heterogeneity defined by *I*^2^ ranges were as follows:

(1) *I*^2^ less than 40%: low heterogeneity.
(2) 30% < *I*^2^ < 60%: potentially moderate heterogeneity.
(3) 50% < *I*^2^ < 90%: potentially substantial heterogeneity.
(4) 75% < *I*^2^ < 100%: considerable heterogeneity.

### 2.9. Assessment of reporting biases

Reporting bias was assessed using a funnel plot. If 10 or more trials were included in a meta-analysis, we examined the funnel plot of the analysis. Generally, if the funnel plot shows a symmetric inverted funnel shape, publication bias is unlikely. In contrast, an asymmetric funnel plot on visual assessment indicated major reporting biases.

### 2.10. Data synthesis

When the *I*^2^ value is 50% or less, we used a fixed-effects model to pool the meta-analysis; for *I*^2^ values greater than 50%, we used a random-effects model to pool the meta-analysis (29).

### 2.11. Subgroup analysis and investigation of heterogeneity

Considering the differences in EA and medication protocols among the trials, we planned to conduct subgroup analyses based on different EA characteristics (acupoints, current frequency, waveform, and retaining time), antidepressant medication characteristics (classes, dosage, and duration), and therapy protocols. We also planned to conduct subgroup analyses of different time points (at the end of week 2, 4, and 6 of treatment).

### 2.12. Sensitivity analysis

We planned to conduct sensitivity analysis by pooling the data after excluding trials that met the following criteria:

(1) High overall ROB.
(2) High ROB in more than two domains.
(3) Dropout rate > 20%.

### 2.13. Evidence tables

The quality evaluation of outcome measures in the meta-analysis was presented in the GRADE system. The GRADE system included six domains: study design, ROB, inconsistency, indirectness, imprecision, and other considerations. On the basis of the domain assessment, the certainty of each meta-analysis was graded as high, moderate, low, and very low.

## 3. Results

### 3.1. Description of studies

#### 3.1.1. Results of the search

**FIGURE 1:**
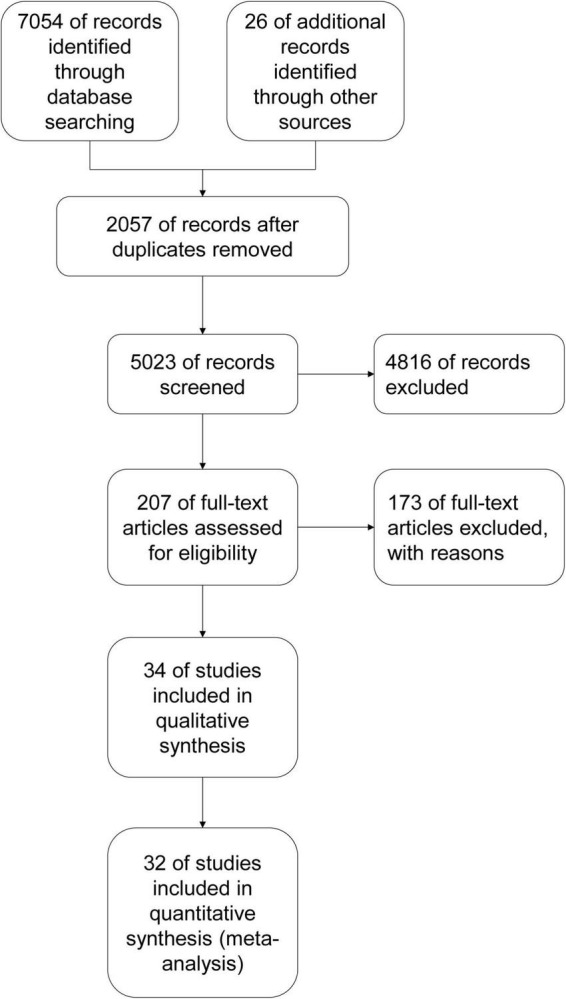
Study flow diagram.

A total of 7,080 article records were retrieved [electronic searches: 7,054 records; other resources: 26 records (30–63)]. After excluding 2,057 duplicates, two independent authors screened and read the remaining article records with an extra author serving as a referee for differences in assessments. The remaining 5,023 records were screened on the basis of their titles and abstracts, and 4,816 records were excluded in this step. The full text of the remaining 207 article records was reviewed, of which 173 were excluded. A total of 34 article records were included in qualitative synthesis, of which 32 were included in quantitative synthesis (meta-analysis) (Figure 1).

#### 3.1.2. Included studies

In the 34 trials included, the number of participants ranged from 20 (34) to 477 (63), with a total of 2,988 participants and a median of 88 participants.

The diagnoses in these trials were primary depression (26 trials), post-stroke depression [4 trials, (30, 33, 36, 42)], postpartum depression [1 trial, (34)], post-schizophrenic depression [1 trial, (44)], PCOS with depression [1 trial, (62)], and depression with insomnia [1 trial, (60)]. With regard to the diagnostic criteria for depression, 12 trials adopted the DSM-III, IV, or V, seven adopted the Chinese Classification and Diagnostic Criteria of Mental Disorders (CCMD)-unknown version or 3, and seven adopted the ICD-9 or 10. Three trials adopted combinations of two criteria (36, 46, 52). Three trials recruited participants based on HAMD or SDS scores (42, 58, 62). Two used criteria reported in books or conferences (33, 47).

The average age of the patients ranged from 30.5 to 72 years. Two trials recruited only female patients (34, 62), and 32 included patients with no sex restriction. Thirty-two trials were conducted in China and three in Hong Kong (34, 35, 59). With regard to interventions, 19 trials adopted 2-arm designs and 15 adopted 3-arm designs. The number of acupoints used in each trial ranged from 2 to 18. GV20, GV29, SP6, PC6, and HT7 were the top five acupoints used. The antidepressant medications used in these trials included SSRIs (23 trials) and tricyclic antidepressants (TCAs; five trials). Six trials did not specify the actual classes of antidepressant medication used. Twenty trials lasted 6 weeks, four trials lasted 8 weeks, four trials lasted 4 weeks, two trials lasted 5 weeks, one trial lasted 10 days, one trial lasted 3 weeks, and two trials lasted more than 8 weeks. Follow-up assessments were described in 10 trials.

All trials assessed the severity of depression at baseline. Most trials adopted HAMD (31 trials), while two trials used the SDS as the primary outcome (54, 62), and one trial used checklist 90 (31) (Table 1 and Supplementary Table 1).

**TABLE 1:** Characteristics of the included trials.

No.	References	Methods	Location	Diagnosis	Method of diagnosis	Group	Age (yr)	Sex (male/ female)	Intervention duration	Frequency of treatment	Time points for assessment	Outcomes	Dropout rate and others
1	(30)	Parallel-arm RCT; Single-center; Single-blind (patients)	China	PSD.	**Stroke:** Guidelines for the diagnosis and treatment of acute ischemic stroke. **Depression:** DSM-V.	EA	67.8 ± 10.91	20/13	4 weeks	3 times a week.	0 week; 2 weeks; 4 weeks; 8 weeks (follow-up).	HAMD-24; SDS; NIHSS; BI; Depression scale of traditional Chinese medicine; AEs.	12.30%
						Non-invasive acupuncture control	66.7 ± 11.42	18/14	4 weeks	3 times a week.			
2	(31)	RCT; Multi-center	China	Primary unipolar depression.	ICD-10.	Paroxetine	35.37 ± 11.37	14/21	6 weeks	Sid.	0 week; 2 weeks; 4 weeks; 6 weeks; 10 weeks (follow-up).	SCL-90.	9.52%
						MA + Paroxetine	32.11 ± 9.38	14/21	6 weeks	**Paroxetine:** Sid; **MA:** every other day, 3 times a week.			
						EA + Paroxetine	31.89 ± 8.81	15/20	6 weeks	**Paroxetine:** Sid; **EA:** every other day, 3 times a week.			
3	(32)	RCT; Single-center	China	Liver-qi stagnation and spleen deficiency in the elderly with post-stroke depression.	**Stroke:** Diagnostic efficacy evaluation criteria for stroke; Diagnostic essentials of various cerebrovascular diseases. **Depression:** Diagnostic efficacy standard of TCM disease and syndrome; CCMD.	Stomach acupuncture (manual manipulation)	72 ± 8	8/11	6 weeks	Once every other day for 21 times.	0 week; 2 weeks; 4 weeks; 6 weeks.	HAMD-/; SDS; SDSS; AEs.	0%
						EA	69 ± 7	9/10	6 weeks	Once every other day for 21 times.			
						Basic treatment control	69 ± 6	10/9	6 weeks	Sid.			
4	(33)	RCT; Single-center	China	PSD	Diagnostic criteria of 4th National Conference on cerebrovascular disease; Diagnostic criteria for Post Stroke Mood Disorders in *Neurorehabilitation* (CT/MRI).	EA	54–78	20/16	8 weeks	5 times a week.	0 week; 8 weeks; 6 months (follow-up).	HAMD-/; AEs.	0%
						Fluoxetine	58–72	18/18	8 weeks	Sid.			
5	(34)	RCT; Multi-center; Single-blind (patients and assessors)	Hong Kong SAR, China	Postpartum depression	DSM-IV.	EA	35.3 ± 4.7	0/20	4 weeks	Twice a week, at least 2 days apart.	0 week; 2 weeks; 4 weeks; 8 weeks (follow-up).	HAMD-17; EPDS; HADS; CGI; Sheehan Disability Scale; CTRS; AEs.	30%; Small sample size and high dropout rate.
						Non-invasive acupuncture control	34.4 ± 2.2		4 weeks	Twice a week, at least 2 days apart.			
6	(35)	RCT; Single-center; Single-blind (patients and assessors)	Hong Kong SAR, China	Depression with insomnia	DSM-IV.	EA + antidepressants	48.8 ± 9.9	14/46	4 weeks	**Antidepressants: /**; **EA:** 3 times a week.	0 week; 1 week; 5 weeks.	SE; ISI; PSQI; HAMD-17; HAMA; HADS; SSI; Sheehan Disability Scale; MFI; ESS; SF-36; AEs.	10.67%
						Sham acupoints acupuncture + antidepressants	50.9 ± 9.5	14/46	4 weeks	**Antidepressants: /**; **Sham acupoints acupuncture:** 3 times a week.			
						Non-invasive acupuncture + antidepressants	47.4 ± 9.5	3/27	4 weeks	**Antidepressants: /**; **Non-invasive acupuncture:** 3 times a week.			
7	(36)	RCT; Single-center	China	PSD	**Stroke:** Diagnostic criteria revised at the 4th National Conference on cerebrovascular disease (1995); Stroke diagnosis by CT or MRI. **Depression:** CCMD-3; DSM-IV.	EA	58.4 ± 9.6	25/13	4 weeks	Once a day, 10 times as a course of treatment, a total of 3 courses of treatment.	0 day; 4 weeks (30 days).	HAMD-/; SDS; Serum 5-HT.	/
						MA	59.21 ± 7.56	23/13	4 weeks	Once a day, 10 times as a course of treatment, a total of 3 courses of treatment.			
						Fluoxetine	56.61 ± 8.21	19/15	4 weeks	Sid.			
8	(37)	RCT; Single-center	China	Depression	CCMD-3; Diagnostic efficacy standard of TCM diseases.	Fluoxetine	39.47 ± 11.20	8/17	6 weeks	Sid.	0 week; 1 week; 2 weeks; 3 weeks; 4 weeks; 5 weeks; 6 weeks.	HAMD-/; SDS; TESS; AEs.	6.67%
						EA	40.42 ± 10.71	9/16	6 weeks	Once a day, 5 times a week, 2 days off for the next week of treatment.			
						Fluoxetine + EA	38.17 ± 11.31	8/17	6 weeks	**Fluoxetine:** Sid; **EA:** once a day, 5 times a week, 2 days off for the next week of treatment.			
9	(38)	RCT; Single-center; Blind method used (assessors)	China	Depression	ICD-10.	Fluoxetine	35 ± 8	6/49	6 weeks	Sid.	0 week; 6 weeks.	HAMD-17; MRI; AEs.	6.67%
						EA + fluoxetine			6 weeks	**Fluoxetine:** Sid; **EA:** once a day, 5 times a week, 2 days off for the next week of treatment.			
10	(39)	RCT; Single-center	China	First episode mild to moderate depression	DSM-V.	MA	39.0 (35.0, 46.5) statistical meaning unknown	8/17	6 weeks	Every other day, 3 times a week.	0 week; 6 weeks; 6 months (follow-up)	HAMD-/; SDS.	10%
						EA	37.0 (32.0, 41.5) statistical meaning unknown	8/17	6 weeks	Every other day, 3 times a week.			
11	(40)	RCT; Single-center; Blind method used (investigators, assessors, data collectors, and statisticians)	China	Comorbidity of anxiety and depression (CAD)	CCMD-3.	rTMS	40.3 ± 13.0	13/32	10 days	Once a day, 10 times in total.	0 day; 5 days; 10 days.	HAMD-/; HAMA.	23.5%
						EA + rTMS	35.4 ± 9.1	13/27	10 days	Once a day, 10 times in total.			
12	(41)	RCT; Single-center	China	Depression	CCMD-3.	EA	49.19 ± 13.46	15/17	6 weeks	Once a day.	0 week; 6 weeks.	HAMD-24; Onset time; Effective time.	/
						Fluoxetine/ paroxetine	47.00 ± 13.08	10/14	6 weeks	Sid.			
13	(42)	RCT; Single-center	China	PSD	Diagnostic essentials of various cerebrovascular diseases revised at the fourth national cerebrovascular disease academic conference in 1996.	Fluoxetine	66.42 ± 6.25	5/5	8 weeks	Sid.	0 week; 2 weeks; 4 weeks; 8 weeks.	HAMD-17; SPECT.	0%
						EA	62.56 ± 6.85	4/7	8 weeks	Once a day, 5 days a week.			
14	(43)	RCT; Single-center; Blind method used (assessors and statisticians)	China	Moderate depression	DSM-IV.	Antidepressants	38.75 ± 11.45	11/19	8 weeks	**Antidepressants:** /;	0 week; 4 weeks; 8 weeks; 12 weeks (follow-up).	HAMD-24; Urinary metabolites.	11.67%
						EA + antidepressants	40.3 ± 10.99	11/19	8 weeks	**Antidepressants:** /; **EA:** once every other day, 3 times a week.			
15	(44)	RCT; Single-center	China	Post schizophrenic depression	ICD-10.	EA + sertraline	29.6 ± 11.2	16/14	6 weeks	**Sertraline:** Sid; **EA:** once every other day, 3 times a week.	0 week; 1 week; 2 weeks; 4 weeks; 6 weeks.	GAS; HAMD-17; AEs.	8.33%
						Sertraline	29.2 ± 10.5	17/13	6 weeks	Sid.			
16	(45)	RCT; Single-center	China	Depression	DSM-III.	EA	39	15/12	5 weeks	Once a day except Sunday.	0 week; 1 week; 2 weeks; 3 weeks; 4 weeks; 5 weeks.	HAMD-24; CGI; SERS (Asberg).	0%
						Amitriptyline	35	6/14	5 weeks	Tid.			
17	(46)	RCT; Multi-center; Single-blind (medication placebo)	China	Depression	National diagnostic criteria for manic depressive disorder; CCMD; ICD-9.	EA + placebo	32 (mini- mum–maxi- mum: 17–64)	109/132	6 weeks	**Placebo:** /; **EA:** once a day.	0 week; 1 week; 2 weeks; 3 weeks; 4 weeks; 5 weeks; 6 weeks.	HAMD-/; CGI; SERS (Asberg); AEs; Biochemical test; Electrophysiological examination.	6.50%
						Amitriptyline			6 weeks	/.			
18	(47)	RCT; Multi-center	China	Depressive psychosis	Criteria presented at the Huangshan Symposium on manic-depression; Handbook of epidemiological investigation mental illness in China.	EA	36	32/22	5 weeks	Once a day.	0 week; 1 week; 2 weeks; 3 weeks; 4 weeks; 5 weeks; 6 weeks.	HAMD-24; CGI; SERS (Asberg).	/
						Amitriptyline		19/28	5 weeks	Tid.			
19	(48)	Parallel RCT; Multi-center; Blind method used (statisticians)	China	Mild or moderate depression	ICD-10.	Paroxetine	40.52 ± 14.21	19/10	6 weeks	Sid.	0 week; 1 week; 2 weeks; 4 weeks; 6 weeks.	HAMD-17; SERS; CGI; AEs.	3.64%
						EA	46.27 ± 13.13	17/9	6 weeks	Once every other day, 3 times a week.			
20	(49)	RCT; Multi-center; Blind method used (statisticians)	China	Depression	ICD-10.	Paroxetine	35.58 ± 10.62	31/34	6 weeks	Sid.	0 week; 1 week; 2 weeks; 4 weeks; 6 weeks.	HAMD-17; SDS; SERS; AEs.	2.05%
						EA + paroxetine	34.03 ± 10.60	28/34	6 weeks	**Paroxetine:** Sid; **EA:** once every other day, 3 times a week.			
						EA	33.2 ± 9.0 36.58 ± 10.9	23/41	6 weeks	Once every other day, 3 times a week.			
21	(50)	RCT; Single-center; Single-blind (patients and assessors)	China	Depression	DSM-IV.	Sham acupoints acupuncture + fluoxetine	33.9 ± 12.4	81 (sex not covered in the original text)	6 weeks	**Fluoxetine:** Sid; **Sham acupoints acupuncture:** every weekend.	0 week; 6 weeks.	HAMD-24; Serum G protein.	/
						EA + placebo	30.8 ± 10.9		6 weeks	**Placebo:** Sid; **EA:** every weekday.			
						Sham acupoints acupuncture + placebo	30.5 ± 12.0		6 weeks	**Placebo:** Sid; **Sham acupoints acupuncture:** every weekday.			
22	(51)	RCT; Single-center; Single-blind (patients and assessors)	China	Depression	DSM-IV.	EA + placebo	30 ± 11	13/19	6 weeks	**Placebo:** Sid; **EA:** 3 times a week.	0 week; 1 week; 2 weeks; 3 weeks; 4 weeks; 5 weeks; 6 weeks.	Serum cytokine; HAMD-24 (21 in the article); CGI.	12.63%
						Sham acupoints acupuncture + fluoxetine	34 ± 13	13/18	6 weeks	**Fluoxetine:** Sid; **Sham acupoints acupuncture:** 3 times a week.			
						Sham acupoints acupuncture + placebo	30 ± 12	13/19	6 weeks	**Placebo:** Sid; **Sham acupoints acupuncture:** 3 times a week.			
23	(52)	RCT; Multi-center	China	Depression	CCMD-3; DSM-IV.	EA1	35.9 ± 14.5	13/15	6 weeks	Once a day, 5 days a week, 2 days off on weekends.	0 week; 2 weeks; 4 weeks; 6 weeks.	HAMD-24; Serum IL-1β, IL-6, and TNF-α.	6.67%
						EA2	41.1 ± 11.5	5/23	6 weeks	Once a day, 5 days a week, 2 days off on weekends.			
						Fluoxetine	39.1 ± 13.2	3/25	6 weeks	Sid.			
24	(53)	RCT; Single-center; Blind method used (assessors of GDNF)	China	Depression	DSM-IV.	EA1	43.10 ± 13.86	8/12	6 weeks	Once a day, 5 times a week.	0 week; 2 weeks; 4 weeks; 6 weeks.	HAMD-24; Serum GDNF.	18.67%
						EA2	42.56 ± 10.70	3/13	6 weeks	Once a day, 5 times a week.			
						Fluoxetine	40.72 ± 12.80	3/22	6 weeks	Sid.			
25	(54)	RCT; Single-center; Non-blind	China	Depression	DSM-IV.	EA	48.10 ± 13.40	7/17	24 weeks	3 times a week.	0 week; 24 weeks.	MMPI; SDS; SAS; MADRS; AEs.	20%
						Paroxetine	47.10 ± 10.60	8/16	24 weeks	Sid.			
26	(55)	Parallel RCT; Multi-center	China	Mild or moderate depression	ICD-10.	Paroxetine	48 ± 9	6/11	6 weeks	Sid.	0 week; 1 week; 2 weeks; 4 weeks; 6 weeks; 10 weeks (follow-up).	HAMD-17; SERS; WHOQOL-Bref; AEs.	12.50%
						MA + paroxetine	45 ± 12	10/22	6 weeks	**Paroxetine:** Sid; **MA:** once every other day, 3 times a week.			
						EA + paroxetine	47 ± 11	3/20	6 weeks	**Paroxetine:** Sid; **EA:** once every other day, 3 times a week.			
27	(56)	RCT; Single-center (part of multi-center)	China	Depression	CCMD-3.	SSRIs	47.42 ± 8.89	11/19	6 weeks	/	0 week; 1 week; 2 weeks; 4 weeks; 6 weeks.	HAMD-17.	0%
						MA + SSRIs	48.01 ± 8.16	10/15	6 weeks	**SSRIs:** /; **MA:** once every other day, 3 times a week.			
						EA + SSRIs	47.54 ± 8.03	7/13	6 weeks	**SSRIs:** /; **EA:** once every other day, 3 times a week.			
28	(57)	RCT; Single-center; Blind method used (assessors)	China	Depression	CCMD.	EA	38 ± 5	10/9	30 times (possibly 6 weeks)	Once a day.	0 week; 1 week; 2 weeks; 3 weeks; 4 weeks; 5 weeks; 6 weeks.	HAMD-/; CGIS.	0%
						Amitriptyline	36 ± 8	3/8	6 weeks	Tid.			
29	(58)	RCT; Single-center	China	Mental depression	Clinical criteria for diagnosis of manic depression.	EA	22–57	17/24	6 weeks	6 times a week, followed by 1 day off;	0 week; 1 week; 2 weeks; 3 weeks; 4 weeks; 5 weeks; 6 weeks.	HAMD-24; EEG.	0%
						Amitriptyline			6 weeks	/.			
30	(59)	Parallel RCT; Single-center; Single-blind (patients and assessors)	Hong Kong SAR, China	Depression	DSM-IV; With insomnia complaint.	EA + antidepressants	47.5 ± 8.5	6/20	3 weeks	**Antidepressants:** /; **EA:** 3 times a week.	0 week; 1 week; 4 weeks (follow-up).	ISI; PSQI; Sleep diary; Actigraphy measures; HAMD-17; AEs.	9%
						Sham acupoint acupuncture + antidepressants	46.7 ± 9.7	7/19	3 weeks	**Antidepressants:** /; **Sham acupoints acupuncture:** 3 times a week.			
						Non-invasive acupuncture + antidepressants	50.1 ± 9.1	3/23	3 weeks	**Antidepressants:** /; **Non-invasive acupuncture:** 3 times a week.			
31	(60)	RCT; Single-center; Single-blind (patients and assessors)	China	Depression with insomnia	DSM-IV.	EA + antidepressants	47.30 ± 14.89	11/19	8 weeks	**Antidepressants:** /; **EA:** 3 times a week.	0 week; 4 weeks; 8 weeks; 12 weeks (follow-up).	PSQI; SE; TST; SA; HAMD-17; SDS; HAMA; AEs.	14.44%
						Sham acupoint acupuncture + antidepressants	49.80 ± 15.13	10/20	8 weeks	**Antidepressants:** /; **Sham acupoints acupuncture:** 3 times a week.			
						Non-invasive acupuncture + antidepressants	46.77 ± 15.57	11/19	8 weeks	**Antidepressants:** /; **Non-invasive acupuncture:** 3 times a week.			
32	(61)	RCT; Single-center	China	Depression	CCMD-3.	Paroxetine	37.1 ± 10.2	9/11	6 weeks	Sid.	0 week; 1 week; 2 weeks; 4 weeks; 6 weeks.	HAMD-17; TESS; Routine blood, urine test; ECG.	0%
						EA + paroxetine	36.6 + 9.7	12/10	6 weeks	**Paroxetine:** Sid; **EA:** once a day, 6 times a week.			
33	(62)	RCT; Single-center	China	PCOS with mild anxiety/depression	Rotterdam PCOS diagnostic criteria; With mild anxiety/depression.	Lifestyle intervention	28 ± 3	0/40	4 months	1 month is a course of treatment, a total of 4 courses of treatment.	0 month; 4 months (16 weeks).	BMI; SAS; SDS; PCOSQ; Hairiness score; Serum sex hormone; AEs.	13.04%
						EA + lifestyle intervention	29 ± 2		4 months	**Lifestyle intervention:** at least 3 times a day; 1 month is a course of treatment, a total of 4 courses of treatment; **EA:** once every other day, 3 times a week.			
34	(63)	RCT; Multi-center; Single-blind (assessors)	China	Depression	ICD-10.	SSRIs	41.76 ± 12.85	57/99	6 weeks	Sid.	0 week; 1 week; 2 weeks; 4 weeks; 6 weeks; 10 weeks (follow-up).	HAMD-17; SDS; CGI; SERS; AEs.	20%
						MA + SSRIs	41.42 ± 12.53	56/105	6 weeks	**SSRIs:** Sid; **MA:** 3 times a week.			
						EA + SSRIs	41.18 ± 12.00	52/108	6 weeks	**SSRIs:** Sid; **EA:** 3 times a week.			

/, not covered in the original text; 5-HT, 5-hydroxytryptamine; AE, adverse event; BI, Barthel Daily Living Index; BMI, body mass index; CCMD, Chinese Classification and Diagnostic Criteria of Mental Disorders; CGI, Clinical Global Impression Scale; CGIS, Clinical Global Impressions Scale; CTRS, Credibility of Treatment Rating Scale; DSM, Diagnostic and Statistical Manual of Mental Disorders; EA, electroacupuncture; ECG, electrocardiograph; EEG, electroencephalogram; EPDS, Edinburgh Postpartum Depression Scale; ESS, Epworth Sleepiness Scale; GAS, Global Assessment Scale; GDNF, glial cell-derived neurotrophic factor; HADS, Hospital Anxiety and Depression Scale; HAMA, Hamilton Anxiety Rating Scale; HAMD, Hamilton Depression Rating the Scale; ICD, International Classification of Diseases; MA, manual acupuncture; MADRS, Montgomery–Asberg Depression Rating Scale; MFI, multidimensional fatigue inventory; MMPI, Minnesota Multiphasic Personality Inventory; NIHSS, National Institutes of Health Stroke Scale; PCOS, polycystic ovary syndrome; PSD, post stroke depression; PCOSQ, Polycystic Ovary Syndrome Quality of Life scale; PSQI, Pittsburgh Sleep Quality Index; RCTs, randomized controlled trials; SA, sleep awake times; SAS, Self-Rating Anxiety Scale; SCL-90, Symptom Checklist-90; SDS, Self-Rating Depression Scale; SDSS, Social Disability Screening Schedule; SE, sleep efficiency; SERS, Side Effects Rating Scale; SF-36, 36-item Short Form Health Survey; SSI, Somatic Symptom Inventory; TESS, Treatment Emergent Symptoms Scale; TCM, Traditional Chinese Medicine; TST, total sleep time; WHOQOL-BREF, World Health Organization Quality of Life.

#### 3.1.3. Risk of bias in included studies

**FIGURE 2:**
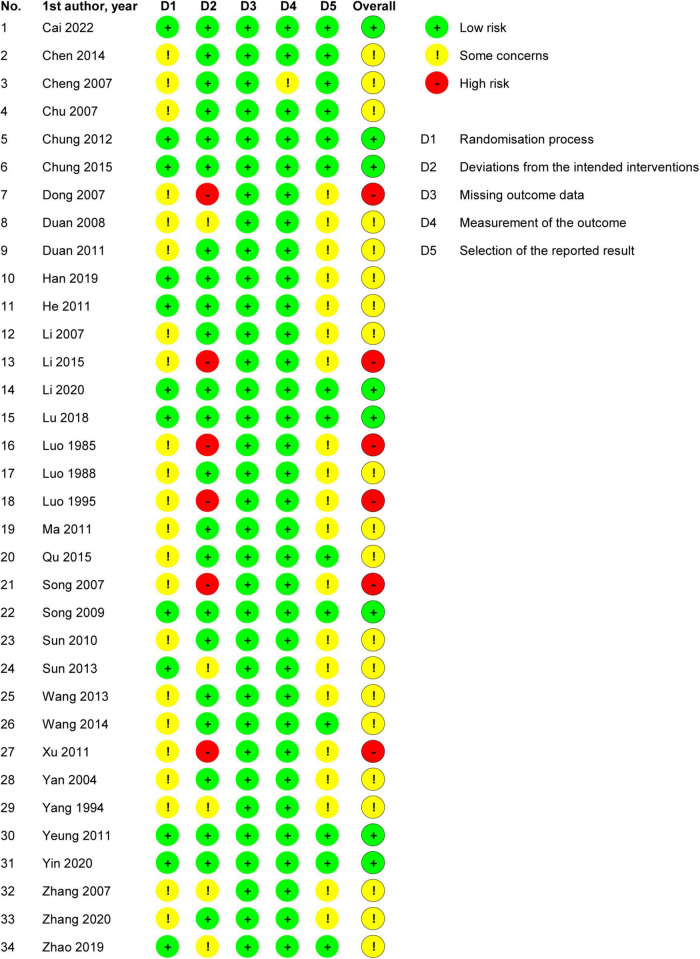
Risk of bias summary: review authors’ judgments about each risk of bias item for each included study.

Two independent authors evaluated the ROB of the included trials. For the randomization process, 22 trials were rated as showing some concerns in ROB, while the rest were rated as showing low ROB. For deviations from intended interventions, six trials were rated as showing high ROB, five were rated as showing some concerns in ROB, and the rest were rated as showing low ROB. For missing outcome data, all trials were rated as showing low ROB. For measurement of the outcome, one trial was rated as showing some concerns in ROB, while the rest were rated as showing low ROB. For selection of the reported result, 20 trials were rated as showing some concerns in ROB, and the rest were rated as showing low ROB. Overall, six trials were rated as showing high ROB, 20 as showing some concerns in ROB, and the rest were rated as showing low ROB (Figure 2).

**FIGURE 3:**
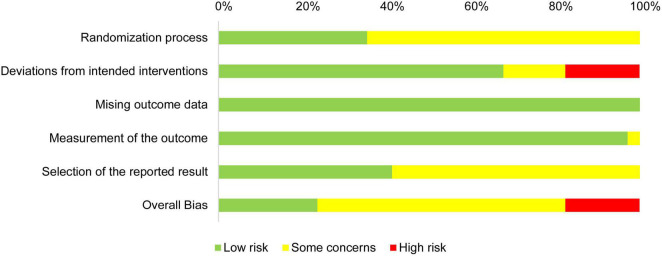
Risk of bias graph: review authors’ judgments about each risk of bias item presented as percentages across all included studies.

In summary, the overall ROB and the ROBs for the randomization process, deviations from intended interventions, and selection of the reported result were medium, while the ROBs for missing outcome data and measurement of the outcome were low (Figure 3).

### 3.2. Effects of interventions

#### 3.2.1. EA vs. antidepressants

##### 3.2.1.1. Severity of depression at the end of treatment

**FIGURE 4:**
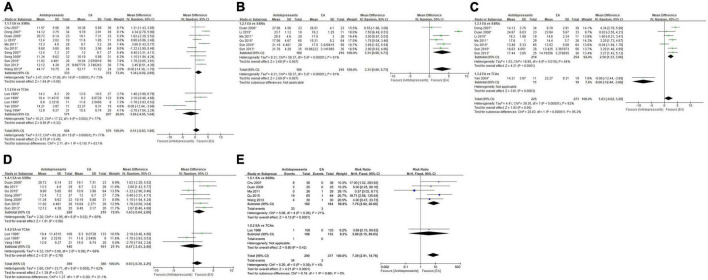
Forest plot of comparison: EA vs. antidepressants. **(A)** Severity of depression at the end of treatment. **(B)** Severity of depression at week 2 during treatment. **(C)** Severity of depression at week 4 during treatment. **(D)** Severity of depression at week 6 during treatment. **(E)** Adverse events. ^1^HAMD; ^2^SDS.

At the end of treatment, very-low-quality evidence (Table 2.1) showed that the overall depression severity of EA was similar to that of antidepressants [MD, 0.51; 95% CI, −0.83 to 1.86; 16 trials, 1,083 participants; Tau^2^ = 5.17; Chi^2^ = 65.28, df = 15 (*P* < 0.00001); *I*^2^ = 77%]. In comparisons with different antidepressants, the depression severity of EA was almost similar to that of SSRIs [MD, 1.34; 95% CI, −0.02 to 2.69; 11 trials, 705 participants; Tau^2^ = 3.47; Chi^2^ = 37.00, df = 10 (*P* < 0.0001); *I*^2^ = 73%] and similar to that of TCAs [MD, −1.66; 95% CI, −4.95 to 1.64; 5 trials, 378 participants; Tau^2^ = 10.21; Chi^2^ = 17.22, df = 4 (*P* = 0.002); *I*^2^ = 77%] (Figure 4A).

**TABLE 2.1:** GRADE evidence table of the main comparison. Electroacupuncture (EA) compared to antidepressants for depression.

	Certainty assessment						Number of patients		Effect		Certainty	Importance
**Number of studies**	**Study design**	**Risk of bias**	**Inconsistency**	**Indirectness**	**Imprecision**	**Other considerations**	**EA**	**Anti- depressants**	**Relative (95% CI)**	**Absolute (95% CI)**		
**EA vs. antidepressants in severity of depression at the end of treatment**												
16	Randomized trials	Serious^a^	Serious^b^	Not serious	Not serious	Publication bias strongly suspected^c^	504	579	–	MD 0.51 (–0.83 to 1.86)	⨁◯◯◯ Very low	Important
**EA vs. antidepressants in severity of depression at week 2 during treatment**												
6	Randomized trials	Not serious	Serious^b^	Not serious	Serious^d^	Publication bias strongly suspected^c^	180	216	–	MD 3.31 (0.9 to 5.73)	⨁◯◯◯ Very low	Critical
**EA vs. antidepressants in severity of depression at week 4 during treatment**												
8	Randomized trials	Serious^a^	Serious^b^	Not serious	Not serious	Publication bias strongly suspected^c^	225	273	–	MD 1.63 (–0.02 to 3.29)	⨁◯◯◯ Very low	Critical
**EA vs. antidepressants in severity of depression at week 6 during treatment**												
10	Randomizsed trials	Serious^a^	Not serious	Not serious	Not serious	Publication bias strongly suspected^c^	369	380	–	MD 0.93 (–0.39 to 2.25)	⨁⨁◯◯ Low	Critical
**EA vs. antidepressants in adverse events**												
6	Randomized trials	Not serious	Not serious	Not serious	Not serious	Publication bias strongly suspected^c^	34/290 (11.7%)	3/317 (0.9%)	**RR 7.39** (2.91 to 18.76)	–	⨁⨁⨁◯ Moderate	Critical

CI, confidence interval; MD, mean difference; RR, risk ratio.

^a^More than one study had high overall risk of bias.

^b^Considerable heterogeneity (*I*^2^ > 75%).

^c^All studies are from the same region/country.

^d^Total event number less than 400.

##### 3.2.1.2. Severity of depression at the end of week 2 during treatment

At the end of week 2 during treatment, very-low-quality evidence (Table 2.1) showed that EA had less overall depression severity to antidepressants [MD, 3.31; 95% CI, 0.90 to 5.73; 6 trials, 396 participants; Tau^2^ = 8.21; Chi^2^ = 58.37, df = 5 (*P* < 0.00001); *I*^2^ = 91%]. As for specific antidepressants, all included trials used SSRIs (Figure 4B).

##### 3.2.1.3. Severity of depression at the end of week 4 during treatment

At the end of week 4 during treatment, very-low-quality evidence (Table 2.1) showed that EA had similar overall depression severity as antidepressants [MD, 1.63; 95% CI, −0.02 to 3.29; 8 trials, 498 participants; Tau^2^ = 4.41; Chi^2^ = 39.30, df = 7 (*P* < 0.00001); *I*^2^ = 82%]. As for specific antidepressants, EA was superior to SSRIs [MD, 2.50; 95% CI, 1.33 to 3.66; 7 trials, 468 participants; Tau^2^ = 1.53; Chi^2^ = 16.88, df = 6 (*P* = 0.010); *I*^2^ = 64%]. Comparison with TCAs could not be performed due to insufficient data (Figure 4C).

##### 3.2.1.4. Severity of depression at the end of week 6 during treatment

At the end of week 6 during treatment, low-quality evidence (Table 2.1) showed that EA had similar overall depression severity as antidepressants [MD, 0.93; 95% CI, −0.39 to 2.25; 10 trials, 749 participants; Tau^2^ = 2.60; Chi^2^ = 23.71, df = 9 (*P* = 0.005); *I*^2^ = 62%]. As for specific antidepressants, EA was barely superior to SSRIs [MD, 1.43; 95% CI, −0.04 to 2.89; 7 trials, 448 participants; Tau^2^ = 2.20; Chi^2^ = 14.95, df = 6 (*P* = 0.02); *I*^2^ = 60%] and similar to TCAs [MD, −0.47; 95% CI, −3.43 to 2.49; 3 trials, 301 participants; Tau^2^ = 4.32; Chi^2^ = 5.80, df = 2 (*P* = 0.06); *I*^2^ = 66%] (Figure 4D).

##### 3.2.1.5. Adverse events

Overall moderate-quality evidence showed (Table 2.1) that EA was associated with a lower adverse event rate than antidepressants [RR, 7.39; 95% CI, 2.91 to 18.76; 6 trials, 607 participants; Chi^2^ = 5.20, df = 5 (*P* = 0.39); *I*^2^ = 4%]. In comparisons with specific antidepressants, EA showed a lower adverse event rate than SSRIs [RR, 7.76; 95% CI, 2.92 to 20.60; 5 trials, 366 participants; Chi^2^ = 5.08, df = 4 (*P* = 0.28); *I*^2^ = 21%]. Comparison with TCAs could not be performed because of insufficient data (Figure 4E).

#### 3.2.2. EA + antidepressants vs. antidepressants

##### 3.2.2.1. Severity of depression at the end of treatment

**FIGURE 5:**
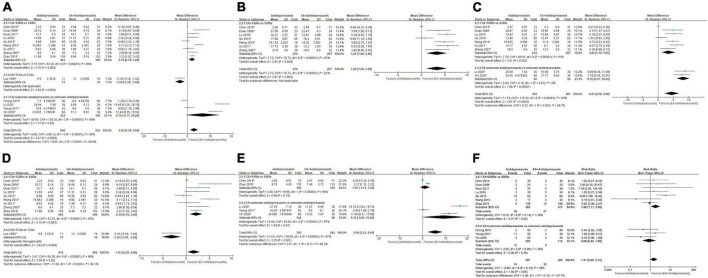
Forest plot of comparison: EA + antidepressants vs. antidepressants. **(A)** Severity of depression at the end of treatment. **(B)** Severity of depression at week 2 during treatment. **(C)** Severity of depression at week 4 during treatment. **(D)** Severity of depression at week 6 during treatment. **(E)** Severity of depression at end of follow-up. **(F)** Adverse events. ^1^HAMD; ^3^checklist 90 depression score.

At the end of treatment, low-quality evidence (Table 2.2) showed that the overall depression severity of EA + antidepressants was less than that of antidepressants alone [MD, 2.99; 95% CI, 1.30 to 4.68; 14 trials, 1198 participants; Tau^2^ = 9.06; Chi^2^ = 209.18, df = 13 (*P* < 0.00001); *I*^2^ = 94%]. In comparisons with different antidepressants, EA + SSRIs was superior to SSRIs [MD, 2.14; 95% CI, 0.78 to 3.49; 9 trials, 799 participants; Tau^2^ = 3.15; Chi^2^ = 53.35, df = 8 (*P* < 0.00001); *I*^2^ = 85%]; however, comparisons with other antidepressants were not possible because of insufficient data (Figure 5A).

**TABLE 2.2:** GRADE evidence table of the main comparison. EA + antidepressants compared to antidepressants for depression.

	Certainty assessment						Number of patients		Effect		Certainty	Importance
**Number of studies**	**Study design**	**Risk of bias**	**Inconsis- tency**	**Indirectness**	**Imprecision**	**Other considera- tions**	**EA + anti- depressants**	**antide- pressants**	**Relative (95% CI)**	**Absolute (95% CI)**		
**EA + antidepressants vs. antidepressants in severity of depression at the end of treatment**												
14	Randomized trials	Serious^a^	Serious^b^	Not serious	Not serious	None	646	552	–	MD 2.99 (1.3 to 4.68)	⨁⨁◯◯ Low	IMPORTANT
**EA + antidepressants vs. antidepressants in severity of depression at week 2 during treatment**												
7	Randomized trials	Not serious	Serious^b^	Not serious	Not serious	Publication bias strongly suspected^c^	214	207	–	MD 3.26 (1.06 to 5.46)	⨁⨁◯◯ Low	CRITICAL
**EA + antidepressants vs. antidepressants in severity of depression at week 4 during treatment**												
9	Randomized trials	Not serious	Serious^b^	Not serious	Not serious	Publication bias strongly suspected^c^	304	267	–	MD 4.47 (2.08 to 6.86)	⨁⨁◯◯ Low	CRITICAL
**EA + antidepressants vs. antidepressants in severity of depression at week 6 during treatment**												
10	Randomized trials	Serious^a^	Serious^b^	Not serious	Not serious	Publication bias strongly suspected^c^	414	406	–	MD 1.63 (0.29 to 2.96)	⨁◯◯◯ Very low	CRITICAL
**EA + antidepressants vs. antidepressants in severity of depression at the end of follow-up**												
5	Randomized trials	Not serious	Serious^b^	Not serious	Not serious	None	310	242	–	MD 5.56 (2.21 to 8.9)	⨁⨁⨁◯ Moderate	IMPORTANT
**EA + antidepressants vs. antidepressants in adverse events**												
10	Randomized trials	Not serious	Not serious	Not serious	Not serious	None	54/567 (9.5%)	30/489 (6.1%)	**RR 1.51** (0.99 to 2.31)	–	⨁⨁⨁⨁ High	CRITICAL

CI, confidence interval; MD, mean difference; RR, risk ratio.

*^a^*More than one study had high overall risk of bias.

*^b^*Considerable heterogeneity (*I*^2^ > 75%).

*^c^*All studies are from the same region/country.

##### 3.2.2.2. Severity of depression at the end of week 2 during treatment

At the end of week 2 during treatment, low-quality evidence (Table 2.2) showed that the overall depression severity of EA + antidepressants was less than that of antidepressants [MD, 3.26; 95% CI, 1.06 to 5.46; 7 trials, 421 participants; Tau^2^ = 7.73; Chi^2^ = 76.72, df = 6 (*P* < 0.00001); *I*^2^ = 92%], and all trials in this comparison adopted SSRIs as the antidepressant medication (Figure 5B).

##### 3.2.2.3. Severity of depression at the end of week 4 during treatment

At the end of week 4 during treatment, low-quality evidence (Table 2.2) showed that the overall depression severity of EA + antidepressants was less than that of antidepressants [MD, 4.47; 95% CI, 2.08 to 6.86; 9 trials, 571 participants; Tau^2^ = 11.74; Chi^2^ = 119.24, df = 8 (*P* < 0.00001); *I*^2^ = 93%]. In comparisons with different antidepressants, EA + SSRIs was superior to SSRIs [MD, 3.42; 95% CI, 1.19 to 5.65; 7 trials, 421 participants; Tau^2^ = 7.70; Chi^2^ = 66.55, df = 6 (*P* < 0.00001); *I*^2^ = 91%]. Comparisons with other antidepressants could not be performed due to insufficient data (Figure 5C).

##### 3.2.2.4. Severity of depression at the end of week 6 during treatment

At the end of week 6 during treatment, very-low-quality evidence (Table 2.2) showed that the overall depression severity of EA + antidepressants was less than that of antidepressants [MD, 1.63; 95% CI, 0.29 to 2.96; 10 trials, 820 participants; Tau^2^ = 3.47; Chi^2^ = 64.26, df = 9 (*P* < 0.00001); *I*^2^ = 86%]. In comparisons with different antidepressants, EA + SSRIs was superior to SSRIs [MD, 2.14; 95% CI, 0.78 to 3.49; 9 trials, 997 participants; Tau^2^ = 3.15; Chi^2^ = 53.35, df = 8 (*P* < 0.00001); *I*^2^ = 85%]. Comparisons with other antidepressants could not be performed due to insufficient data (Figure 5D).

##### 3.2.2.5. Severity of depression at the end of the follow-up period

At the end of the follow-up assessments after the trials, moderate-quality evidence (Table 2.2) showed that the overall depression severity of EA + antidepressants was less than that of antidepressants [MD, 5.56; 95% CI, 2.21 to 8.90; 5 trials, 552 participants; Tau^2^ = 13.10; Chi^2^ = 138.60, df = 4 (*P* < 0.00001); *I*^2^ = 97%]. However, in comparisons with different antidepressants, EA + SSRIs was similar to SSRIs [MD, 1.10; 95% CI, −1.11 to 3.30; 2 trials, 324 participants; Tau^2^ = 2.40; Chi^2^ = 19.86, df = 1 (*P* < 0.00001); *I*^2^ = 95%]. Comparisons with other antidepressants could not be performed due to insufficient data (Figure 5E).

##### 3.2.2.6. Adverse events

Overall high-quality evidence (Table 2.2) showed that EA + antidepressants almost had a lower adverse event rate than antidepressants [RR, 1.51; 95% CI, 0.99 to 2.31; 10 trials, 1,056 participants; Chi^2^ = 14.63, df = 9 (*P* = 0.10); *I*^2^ = 38%]. In comparisons with different antidepressants, EA + antidepressants showed a lower adverse event rate than SSRIs [RR, 1.86; 95% CI, 1.11 to 3.09; 7 trials, 738 participants; Chi^2^ = 9.70, df = 6 (*P* = 0.14); *I*^2^ = 38%]. Comparison of EA + TCAs with other antidepressants could not be performed due to insufficient data (Figure 5F).

#### 3.2.3. EA vs. MA

##### 3.2.3.1. Severity of depression at the end of treatment

At the end of treatment, very-low-quality evidence (Supplementary Table 2) showed that EA had similar depression severity as MA [MD, 2.93; 95% CI, −1.96 to 7.82; 2 trials, 158 participants; Tau^2^ = 16.06; Chi^2^ = 15.94, df = 2 (*P* = 0.0003); *I*^2^ = 87%] (Supplementary Figure 1). Only one trial (32) qualified for the comparison of adverse events, and no adverse events occurred in either group in this trial.

#### 3.2.4. EA + antidepressants vs. MA + antidepressants

##### 3.2.4.1. Severity of depression at the end of treatment

At the end of treatment, moderate-quality evidence (Supplementary Table 3) showed that EA + antidepressants had similar depression severity as MA + antidepressants [MD, 0.18; 95% CI, −0.11 to 0.46; 4 trials, 464 participants; Chi^2^ = 1.48, df = 3 (*P* = 0.69); *I*^2^ = 0%] (Supplementary Figure 2A).

##### 3.2.4.2. Severity of depression at the end of week 2 during treatment

At the end of week 2 during treatment, low-quality evidence (Supplementary Table 3) showed that EA + antidepressants had similar depression severity as MA + antidepressants [MD, 0.13; 95% CI, −0.22 to 0.47; 4 trials, 193 participants; Chi^2^ = 1.22, df = 3 (*P* = 0.75); *I*^2^ = 0%] (Supplementary Figure 2B).

##### 3.2.4.3. Severity of depression at the end of week 4 during treatment

At the end of week 4 during treatment, low-quality evidence (Supplementary Table 3) showed that EA + antidepressants had similar depression severity as MA + antidepressants [MD, 0.01; 95% CI, −0.30 to 0.32; 3 trials, 154 participants; Chi^2^ = 0.91, df = 2 (*P* = 0.64); *I*^2^ = 0%] (Supplementary Figure 2C).

##### 3.2.4.4. Severity of depression at the end of week 6 during treatment

At the end of week 6 during treatment, moderate-quality evidence (Supplementary Table 3) showed that EA + antidepressants had similar depression severity as MA + antidepressants [MD, 0.18; 95% CI, −0.11 to 0.46; 4 trials, 464 participants; Chi^2^ = 1.48, df = 3 (*P* = 0.69); *I*^2^ = 0%] (Supplementary Figure 2D).

##### 3.2.4.5. Adverse events

Overall, moderate-quality evidence (Supplementary Table 3) showed no difference in the rate of adverse events between EA + antidepressants and MA + antidepressants [RR, 0.65; 95% CI, 0.30 to 1.37; 3 trials, 446 participants; Chi^2^ = 2.09, df = 2 (*P* = 0.35); *I*^2^ = 4%]. All the included trials adopted SSRIs as antidepressants (Supplementary Figure 2E).

#### 3.2.5. EA vs. control

##### 3.2.5.1. Severity of depression at the end of treatment

In general, at the end of treatment, low-quality evidence (Supplementary Table 4) showed that EA had less depression severity than control treatment methods [MD, 3.70; 95% CI, 0.38 to 7.01; 5 trials, 241 participants; Tau^2^ = 11.34; Chi^2^ = 20.74, df = 4 (*P* = 0.0004); *I*^2^ = 81%] (Supplementary Figure 3). In comparisons with specific control treatment methods, EA almost showed a superior effect to invasive acupuncture control [MD, 2.59; 95% CI, −0.09 to 5.26; 2 trials, 117 participants; Tau^2^ = 0.73; Chi^2^ = 1.23, df = 1 (*P* = 0.27); *I*^2^ = 19%]. EA showed almost similar depression severity as the non-invasive acupuncture control [MD, 2.76; 95% CI, −5.66 to 11.18; 2 trials, 83 participants; Tau^2^ = 34.33; Chi^2^ = 13.95, df = 1 (*P* = 0.0002); Tau^2^ = 34.33; Chi^2^ = 13.95, df = 1 (*P* = 0.0002); *I*^2^ = 93%]. Comparisons with the waitlist control could not be performed due to insufficient data (Supplementary Figure 3).

#### 3.2.6. EA vs. other therapies

Only two trials compared EA with other therapies, which were lifestyle interventions (62) and rTMS (40). EA + lifestyle intervention showed less depression severity than lifestyle intervention alone. One patient reported an adverse event after EA (62). EA + rTMS also showed less depression severity than rTMS alone with no adverse events (40).

### 3.3. Heterogeneity

The overall heterogeneity among the included trials was substantial. The heterogeneity could be attributed to the following factors:

(1) The patients’ diagnoses included primary, secondary, and comorbid depression.
(2) The average age of the patients varied from 30.5 to 72 years.
(3) Two trials only included female patients.
(4) The duration of the interventions varied from 10 days to 24 weeks.
(5) The control medications varied from SSRI and TCAs to unknown.
(6) The frequency of EA varied from twice a week to five times a week.
(7) The waveform, frequency, intensity, and voltage of the EA current varied across trials.
(8) The scale for evaluation varied from HAMD (−17, −24, unknown version) to SCL-90 depression score.

We planned to conduct subgroup meta-analysis based on differences in EA characteristics, medications, and therapies. However, except for the antidepressant medication, we could not find enough trials to conduct meta-analyses. To minimize the effects of heterogeneity, a random-effects model was used to pool the results of all trials if *I*^2^ > 50%.

### 3.4. Sensitivity analysis

For the sensitivity analysis, we excluded trials with high overall ROBs. However, sensitivity analysis was not performed since very few trials (<5) remained in most comparisons. When trials with high overall ROB, high ROB in more than two domains, or dropout rate > 20% were excluded, comparisons of EA + antidepressants with antidepressants showed no significant difference in the severity of depression at the end of treatment [MD, 5.17; 95% CI, 1.05 to 9.29; 6 trials, 741 participants; Tau^2^ = 25.04; Chi^2^ = 126.74, df = 5 (*P* < 0.00001); *I*^2^ = 96%] and the adverse event rate [RR, 0.95; 95% CI, 0.55 to 1.66; 5 trials, 694 participants; Chi^2^ = 3.57, df = 4 (*P* = 0.47); *I*^2^ = 0%]. Other comparisons and subgroup analyses were not feasible due to the lack of trials.

### 3.5. Reporting bias

**FIGURE 6:**
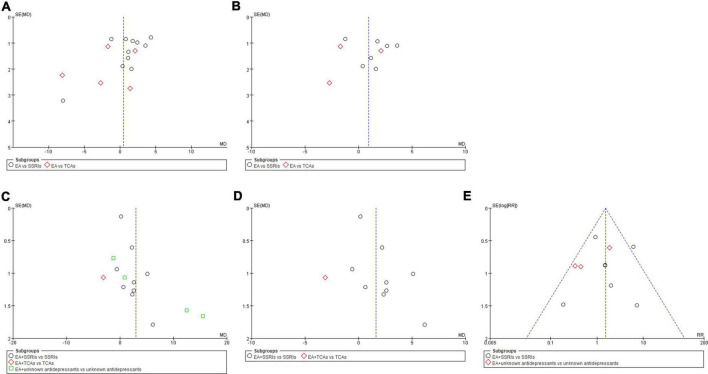
Funnel plots of comparisons. **(A)** EA vs. antidepressants in severity of depression at the end of treatment. **(B)** EA vs. antidepressants in severity of depression at week 6 during treatment. **(C)** EA + antidepressants vs. antidepressants in severity of depression at the end of treatment. **(D)** EA + antidepressants vs. antidepressants in severity of depression at week 6 during treatment. **(E)** EA + antidepressants vs. antidepressants in adverse events.

Funnel plots of comparisons with 10 or more trials were examined. The plots showed a roughly symmetric inverted funnel shape, indicating that major publication bias was unlikely (Figure 6).

## 4. Discussion

A total of 34 trials with 2,988 participants were included, and the data were pooled to perform systematic review and meta-analysis. The 34 trials included both outpatients and inpatients. Although a few trials only included female patients, patients of both sexes were eligible for most trials. Most trials were based in mainland China, while some were conducted in Hong Kong. The treatment acupoints varied across trials, but almost all trials used GV20 and GV29 as intervention acupoints. The comparison groups mainly received antidepressant medication, and none of the studies included treatment control, sham acupuncture, psychology, and physical therapy groups. The majority of antidepressant medications were SSRIs and TCAs. However, some trials did not specify the category of antidepressant medication.

The quality of evidence was generally moderate. Six of the 34 trials were rated as showing high overall ROB, 20 were rated as showing some concerns, and eight were rated as low risk. Blinding-related biases contributed most to the high ROB (the “deviations from the intended interventions” domain), which could be mainly attributed to the nature of EA, which precluded blinding of patients and clinicians administering the treatment. Efforts were made to minimize the bias attributable to patients and assessors.

The ROBs for the randomization process and selection of the reported result were generally medium, mainly due to flaws in the study design and staff arrangement. However, the ROBs for missing outcome data and measurement of the outcome were generally low. We mainly compared the main effects of EA vs. antidepressants and EA + antidepressants vs. antidepressants. Comparisons of EA vs. MA, EA + antidepressants vs. MA + antidepressants, and EA vs. control interventions were also conducted.

Generally, EA showed greater efficacy than SSRIs in week 2 and 4, but not in week 6. Very weak evidence showed that, generally, EA had similar efficacy as TCAs. As for safety, EA showed a lower rate of adverse events than antidepressants, especially SSRIs.

Moderate-quality evidence indicated that EA + antidepressants showed better efficacy than antidepressants alone at the end of treatment, during the treatment and follow-up. EA + SSRIs contributed the most to this finding. Moreover, EA + antidepressants, especially SSRIs, showed a lower adverse event rate than antidepressants alone.

Low-to-moderate-quality evidence also indicated that EA had similar efficacy and safety as MA alone or in combination with antidepressants. Very-low-quality of evidence showed that the efficacies of EA + lifestyle intervention/rTMS were similar to those of lifestyle intervention/rTMS alone at the end of the treatment.

Beside reviewing the included trials, we also evaluated six other published reviews on the effectiveness of EA for depression. We found three meta-analyses addressing EA for depression in 2018. A Cochrane meta-analysis of acupuncture (including EA) for depression (13 trials of EA) (15) yielded low-quality evidence indicating that EA and EA + SSRIs reduce the severity of depression during and at the end of treatment more than SSRIs alone. The efficacy of EA was also similar to invasive control and non-invasive electro-control. As for safety, the findings showed no evidence of an increase in adverse events with EA. Another meta-analysis of the use of EA for post-stroke depression (16) also yielded low-quality evidence (18 trials), indicating that EA and antidepressants have similar efficacy in decreasing the severity of depression. However, EA caused fewer adverse events than antidepressants. Another meta-analysis of acupuncture for postpartum depression included four trials of EA (17); however, a meta-analysis for EA was not conducted because of insufficient data and trials. Notably, the trials included in the latter two studies are mostly from Chinese databases.

In 2020, one systematic review included two trials evaluating the endocrine and immune effects of EA in depression (64). Both studies reported that EA had a positive effect on depression. In 2021, two related reviews were published. One was an update of a meta-analysis of EA for post-stroke depression in 2018 (19 trials) (18), which indicated that in comparison with antidepressants, EA has a similar effect in improving depression symptoms and shows better safety in post-stroke depression patients. Another systematic review focused on the neuroimaging effects of acupuncture (including EA) in depression (10 trials of EA) (65). However, that study did not conduct a meta-analysis of efficacy or neuroimaging data of EA for depression because of the limited number of trials and the fact that most of the trials used pre-post designs. Nevertheless, the study did show that acupuncture affects certain brain regions such as the precuneus, IFG, and ACC.

This meta-analysis included 34 trials. The quality of the evidence is slightly higher comparing with previous reviews due to the greater number of trials. Consistent with the results of previous reviews, we also found low-to-moderate–quality evidence indicating that EA and EA + SSRIs had better efficacy than SSRIs alone in decreasing severity of depression during the treatment. Moreover, EA and EA + SSRIs also caused fewer adverse events than SSRIs. As for other antidepressants, very weak evidence showed that EA and TCAs had similar efficacy, while data for the comparison of EA + TCAs and TCAs were inadequate. Moreover, safety data were insufficient for comparison.

Our findings also yielded low-to-moderate–quality evidence showing that EA and MA had similar efficacy alone or in combination with SSRIs. EA + SSRIs and MA + SSRIs seemed to be equally safe. Moreover, EA had better overall efficacy than control, and single trials showed that EA + lifestyle intervention/rTMS was similar to lifestyle intervention/rTMS alone. Thus, considering the limitations of SSRIs and other antidepressants, including the lack of response and adverse reactions (11), EA may serve as a possible alternative therapy to antidepressants such as SSRIs.

Another limitation of SSRIs is delayed onset of action (12). Since the pooled data indicated that EA or EA + SSRIs decreased the severity of depression during and at the end of treatment more than SSRIs alone during the treatment (2 and 4 weeks), EA may be used to accelerate the action of SSRIs or serve as an alternative for SSRIs in the first few weeks until the effects of SSRIs begin to manifest. Another notable aspect was that MA exerted similar effects as EA alone or in combination with SSRIs. Thus, MA may be an alternative for EA under conditions involving limited equipment.

To our best knowledge, this review presents a comprehensive assessment of the current evidence of the efficacy of EA in depression, and is the largest review on this topic to date. We also compare EA to other therapies (especially SSRIs antidepressants), in early, middle, and later phrases of treatment to examine the rapid-onset effect of EA. We also compared the safety of EA with other therapies. The results could provide new evidence for the supplementary and complementary usage of EA for antidepressants. However, for the efficacy and safety of EA for depression, the quality of the evidence remained at a low-to-moderate level. Although this was better than the previous reviews, more high-quality trials are needed for further confirmation of the current results. One notable matter regarding the trail quality improvement for future review is EA protocol for depression. Our review and other reviews showed substantial heterogeneity between trials. A major cause of this heterogeneity may be the diversity of EA intervention protocols. Thus, standardization and popularization of an EA protocol for depression may be a precondition for future studies.

Besides, we conducted comparison EA to multiple treatments using subgroups comparison. This study could also be done by a network meta-analysis, which could provide more clear results. Although the method we used emphasizes the efficacy of EA, we will also learn to use network meta-analysis in the further research.

Potential biases during the review process may be that non-English databases were not systematically searched because of the generally low quality of trials included in the non-English databases. This may have resulted in exclusion of some eligible trials. We also tried to contact the corresponding author for data if vital details were not published. These missing data may have resulted in biases. Moreover, one of the authors (YH) of this study was an author of one included trial (31). The reviews and ROB evaluations were conducted by other authors.

## 5. Conclusion

These results indicate that the efficacy of EA is no less than that of antidepressant medication. EA also had similar efficacy to MA alone or combined with antidepressants. Moreover, EA + SSRIs was more effective than SSRIs alone throughout the treatment period. In the early period, EA and EA + SSRIs showed a more rapid onset of effects than SSRIs alone. Besides, the safety of EA and EA + SSRIs was superior to that of SSRIs alone. However, the quality of the evidence was still at a low-to-moderate level, and more high-quality trials with standardized EA protocols are needed for further confirmation.

## Data availability statement

The original contributions presented in this study are included in the article/Supplementary material, further inquiries can be directed to the corresponding authors.

## Author contributions

LL: conceptualization and investigation. XC, YL, and RZ: validation. ZZ: methodology and formal analysis. YL, XL, and LL: ROB assessment. YL and XL: selecting the articles and extracting the data. YH: resources, writing – review and editing, project administration, and funding acquisition. XC and RZ: data curation. ZZ and XC: writing – original draft preparation. ZZ and RZ: visualization. LL and YH: supervision. All authors have read and agreed to the published version of the manuscript.

## Footnotes

1
www.crd.york.ac.uk/prospero/display_record.php?RecordID=329143
