# Supplementary material for: Electroacupuncture as a rapid-onset and safer complementary therapy for depression: A systematic review and meta-analysis
Source: Front Psychiatry. 2023 Jan 6;13:1012606. doi: 10.3389/fpsyt.2022.1012606 (PMC9853905; doi:10.3389/fpsyt.2022.1012606)
Supplement: Supplementary file 1 [file Data_Sheet_1.docx]

Supplementary Material

# Supplementary Data

# Search Strategy

Date: March 7th, 2022

1. (Depression) OR (Depressive Symptoms) OR (Depressive Symptom) OR (Symptom, Depressive) OR (Symptoms, Depressive) OR (Emotional Depression) OR (Depression, Emotional) OR (Depressive Disorder) OR (Depressive Disorders) OR (Disorder, Depressive) OR (Disorders, Depressive) OR (Neurosis, Depressive) OR (Depressive Neuroses) OR (Depressive Neurosis) OR (Neuroses, Depressive) OR (Depression, Endogenous) OR (Depressions, Endogenous) OR (Endogenous Depression) OR (Endogenous Depressions) OR (Depressive Syndrome) OR (Depressive Syndromes) OR (Syndrome, Depressive) OR (Syndromes, Depressive) OR (Depression, Neurotic) OR (Depressions, Neurotic) OR (Neurotic Depression) OR (Neurotic Depressions) OR (Melancholia) OR (Melancholias) OR (Unipolar Depression) OR (Depression, Unipolar) OR (Depressions, Unipolar) OR (Unipolar Depressions) OR (Depressive Disorder, Major) OR (Depressive Disorders, Major) OR (Major Depressive Disorders) OR (Major Depressive Disorder) OR (Paraphrenia, Involutional) OR (Involutional Paraphrenia) OR (Involutional Paraphrenias) OR (Paraphrenias, Involutional) OR (Psychosis, Involutional) OR (Involutional Psychoses) OR (Involutional Psychosis) OR (Psychoses, Involutional) OR (Depression, Involutional) OR (Involutional Depression) OR (Melancholia, Involutional) OR (Involutional Melancholia)
2. (electroacupuncture) OR (electric acupuncture) OR (galvanoacupuncture) OR (electro-acupuncture) OR (elektroakupunktura) OR (EA)
3. 1 AND 2

# Supplementary Figures and Tables

# 2.1 Supplementary Tables

**Supplementary Table 1.** Detailed characteristics of the included trials.

| **No.** | **Reference (author, year)** | **1^st^ author, year** | **Methods** | **Location** | **Diagnosis** | **Method of diagnosis** | **Inclusion/Exclusion criteria** | **Group** | **Age (yr)** | **Sex (male/female)** | **Intervention duration** | **Frequency of treatment** | **Treatment protocol** | **Time points for assessment** | **Outcomes** | **Dropout rate and others** |
| --- | --- | --- | --- | --- | --- | --- | --- | --- | --- | --- | --- | --- | --- | --- | --- | --- |
| 1 | (Cai et al., 2022) | Cai 2022 | Parallel-arm RCT;  Single-center;  Single-blind (patients) | China | PSD | **Stroke:**  Guidelines for the diagnosis and treatment of acute ischemic stroke.  **Depression:**  DSM-V. | **Inclusion:**  Diagnosis of PSD;  HAMD-24: 8-35;  40-85 yr.  **Exclusion:**  With severe diseases or other neurological/musculoskeletal disorders affecting functional rehabilitation;  Fear of EA. | EA | 67.8±10.91 | 20/13 | 4 weeks | 3 times a week. | **Acupoints:** GV20, EX-HN1, BL18, SP6, LR3;  **Insertion:** 10-15mm;  **Current:** 2/100Hz (SP6, LR3);  **Retaining time:** 30 min. | 0wk;  2wk;  4wk;  8wk (followk-up). | HAMD-24;  SDS;  NIHSS;  BI;  Depression scale of traditional Chinese medicine;  AEs. | 12.30% |
|  |  |  |  |  |  |  |  | Non-invasive acupuncture control | 66.7±11.42 | 18/14 | 4 weeks | 3 times a week. | Park device with blunted needles that do not penetrate the skin;  Installed with a PG-306 electric pulse generator gives no electrical stimulation. |  |  |  |
| 2 | (Chen et al., 2014) | Chen 2014 | RCT;  Multi-center | China | Primary unipolar depression | ICD-10. | **Inclusion:**  Mild or moderate depressive episodes, at acute phase of depression;  HAMD-/: ≥ 17;  18-60 yr.  **Exclusion:**  Participated in other clinical trials within 4 weeks;  Taking or in washout phase of antidepressants;  Suicidal tendency;  With comorbid systemic diseases, brain diseases, psychosis or illiteracy, thrombocytopenia and hemophilia;  Pregnant, potentially pregnant, lactating, postpartum or menopausal. | Paroxetine | 35.37±11.37 | 14/21 | 6 weeks | Sid. | **Paroxetine:** 1d-2d: 10mg/d; 2d-6w: 20mg/d; | 0wk;  2wk;  4wk;  6wk;  10wk (followk-up). | SCL-90. | 9.52% |
|  |  |  |  |  |  |  |  | MA+Paroxetine | 32.11±9.38 | 14/21 | 6 weeks | **Paroxetine:** Sid;  **MA:** every other day, 3 times a week. | **Paroxetine:** same as the paroxetine group;  **Acupoints:** GV20, GV29, GV14, GV16, GB20, PC6, SP6;  **Insertion:** /;  Manual manipulation;  **Retaining time:** / min. |  |  |  |
|  |  |  |  |  |  |  |  | EA+Paroxetine | 31.89±8.81 | 15/20 | 6 weeks | **Paroxetine:** Sid;  **EA:** every other day, 3 times a week. | **Paroxetine:** same as the paroxetine group;  **Acupoints:** GV20, GV29, GV14, GV16, GB20, PC6, SP6, ST36, HT7, SJ6, GB8;  **Insertion:** /;  **Current:** dilatational wave, 22/15Hz (GV20, GV29);  **Retaining time:** 30 min, twisted once at 15 min for 5-10 seconds. |  |  |  |
| 3 | (Cheng and Tang, 2007) | Cheng 2007 | RCT;  Single-center | China | Liver-qi stagnation and spleen deficiency in the elderly with post-stroke depression | **Stroke:**  Diagnostic efficacy evaluation criteria for stroke;  Diagnostic essentials of various cerebrovascular diseases.  **Depression:**  Diagnostic efficacy standard of TCM disease and syndrome;  CCMD. | **Inclusion:**  Diagnosis of stroke (infarction/hemorrhage) and depression;  Secondary depression after acute attack of stroke for at least 2 weeks;  Mild or moderate depression (HAMD-/: 20-35, SDS: > 41);  60-85 yr;  No history of mental illness, drug abuse and alcohol abuse.  **Exclusion:**  Primary depression, secondary depression of non-stroke causes;  Other antidepressants administration within 2 weeks;  Severe depression (HAMD-/: > 35);  Antidepressants or ethanol allergy;  Needle fainting, infection, ulcer, scar or tumor at the intervention acupoint. | Stomach acupuncture (manual manipulation) | 72±8 | 8/11 | 6 weeks | Once every other day for 21 times. | **Acupoints:** GV12, GV10, GV6, GV4, SP15, ST24, ST26;  **Insertion:** perpendicular insertion (subcutaneous, between adipose layers, above muscle layer;);  **Manual manipulation:** fast insertion, twirling only, no lifting and thrusting, deqi not request;  **Retaining time:** 30 min. | 0wk;  2wk;  4wk;  6wk. | HAMD-/;  SDS;  SDSS;  AEs. | 0% |
|  |  |  |  |  |  |  |  | EA | 69±7 | 9/10 | 6 weeks | Once every other day for 21 times. | **Acupoints:** GV20, GV29, EX-HN1, LR3, HT7, GV24;  **Insertion:** transverse insertion 0.5-1 cun (GV20, GV24, EX-HN1); perpendicular insertion 0.5-1 cun (LR3, HT7);  **Current:** dilatational wave, 4/20 Hz, 35-50V;  **Retaining time:** 30 min. |  |  |  |
|  |  |  |  |  |  |  |  | Basic treatment control | 69±6 | 10/9 | 6 weeks | Sid. | Basic stroke treatment. |  |  |  |
| 4 | (Chu et al., 2007) | Chu 2007 | RCT;  Single-center | China | PSD | Diagnostic criteria of 4^th^ National Conference on cerebrovascular disease;  Diagnostic criteria for Post Stroke Mood Disorders in *Neurorehabilitation* (CT/MRI). | **Inclusion:**  Diagnosis of stroke (CT/MRI);  HAMD-/: score /.  **Exclusion:**  With primary depression, severe mental disorder, aphasia, neurasthenia and severe dementia before stroke. | EA | 54-78 | 20/16 | 8 weeks | 5 times a week. | **Acupoints:** GV20, GV29, GV16, PC6, ST36, LR3, GV26, BL23, GV24, CV6, BL20;  **Insertion:** /;  **Current:** density wave, 6V, 6-8Hz;  **Retaining time:** 30 min. | 0wk;  8wk;  6m (followk-up). | HAMD-/;  AEs. | 0% |
|  |  |  |  |  |  |  |  | Fluoxetine | 58-72 | 18/18 | 8 weeks | Sid. | **Fluoxetine:** 20mg/d; |  |  |  |
| 5 | (Chung et al., 2012) | Chung 2012 | RCT;  Multi-center;  Single-blind (patients and assessors) | Hongkong | Postpartum depression | DSM-IV. | **Inclusion:**  Within 6 months postpartum;  EPDS: ≥12;  Mild depression (HAMD-17: 12-19);  ≥18 yr.  **Exclusion:**  A previous diagnosis of other mental disorders;  Suicidal or infanticidal tendency;  Severe physical diseases;  With heart valve defect, hemorrhagic disease or taking anticoagulant drugs;  Infection or abscess near the intervention acupoints;  Received acupuncture within 12 months before 0w;  Taking herbal or psychotropic drugs, counseling, psychotherapy for depression at 0w or during the study period. | EA | 35.3±4.7 | 0/20 | 4 weeks | Twice a week, at least 2 days apart. | **Acupoints:** GV20, GV29, EX-HN1, GB15, GB8, EX-HN5, ST8, SP6, LR3, HE7, PC6;  **Insertion:** 10-30mm obliquely insertion or perpendicular insertion;  **Current:** 6V, biphasic triangular, brief-pulse, 2Hz;  **Retaining time:** 30 min. | 0wk;  2wk;  4wk;  8wk (followk-up). | HAMD-17;  EPDS;  HADS;  CGI;  Sheehan Disability Scale;  CTRS;  AEs. | 30%;  Small sample size and high dropout rate. |
|  |  |  |  |  |  |  |  | Non-invasive acupuncture control | 34.4±2.2 |  | 4 weeks | Twice a week, at least 2 days apart. | **Acupoints:** same as the EA group;  Streitberger’s placebo needles with blunt needle that will not penetrate skin, held by surgical tape or hair pins and connected to the same electric stimulator using the same stimulation modality. |  |  |  |
| 6 | (Chung et al., 2015) | Chung 2015 | RCT;  Single-center;  Single-blind (patients and assessors) | Hong Kong | Depression with insomnia | DSM-IV. | **Inclusion:**  Previous diagnosis of depression;  Diagnosis of primary insomnia;  Predominant complaint of sleep difficulty associated with distress or functional impairment;  With insomnia ≥ 3 nights/week for at least 3 months;  Insomnia Severity Index (ISI) ≥ 15;  Taken the same antidepressants at fixed doses for at least 12 weeks prior to 0 wk and during the study;  18-70 yr.  **Exclusion:**  HAMD-17: >18;  Apnea hypopnea index ≥ 10;  Periodic limb movement disorder index ≥ 15;  Suicidal tendency (HAMD-17 suicide score ≥ 3);  With psychotic disorders, bipolar disorder, addiction;  Pregnant, potentially pregnant, lactating;  Infection or abscess at the selected acupoints;  With severe diseases;  Taken acupuncture within 12 months;  Taken Chinese herbal medicine or over-the counter drugs for insomnia within 2 weeks;  Taken hypnotics at an increased dose within 4 weeks. | EA+antidepressants | 48.8±9.9 | 14/46 | 4 weeks | **Antidepressants:** /;  **EA:** 3 times a week. | Continue the same type and dosage of antidepressants throughout the study period;  **Acupoints:** Shemmen (auricular point), EX-HN1, Anmian, PC6, HT7, SP6, GV29, GV20 (selection based on expert opinion);  **Insertion:** 2 mm-25 mm, depending on the points selected;  **Current:** constant current, 0.4-ms, square-wave, 4-Hz frequency to the subjects;  **Retaining time:** 30 min. | 0 wkk;  1 wkk;  5 wkk. | SE;  ISI;  PSQI;  HAMD-17;  HAMA;  HADS;  SSI;  Sheehan Disability Scale;  MFI;  ESS;  SF-36;  AEs. | 10.67% |
|  |  |  |  |  |  |  |  | Sham acupoints acupuncture+antidepressants | 50.9±9.5 | 14/46 | 4 weeks | **Antidepressants:** /;  **Sham acupoints acupuncture:** 3 times a week. | Continue the same type and dosage of antidepressants throughout the study period;  **Sham acupoint:** 1 inch lateral to the middle point between HT3 and HT7; 1 inch lateral to LU3; 0.5 inch dorsal to GB39. The middle point between GB8 and ST8; the middle point between ST8 and GB14; the middle point between TE16 and SI17; “ear”, a point on the ear helix, inferior to the apex;  **Insertion:** superficially to avoid deqi;  Other treatment conditions and electrostimulation were the same as the in the acupuncture group. |  |  |  |
|  |  |  |  |  |  |  |  | Non-invasive acupuncture +antidepressants | 47.4±9.5 | 3/27 | 4 weeks | **Antidepressants:** /;  **Non-invasive acupuncture:** 3 times a week. | Continue the same type and dosage of antidepressants throughout the study period;  Streitberger placebo needles were placed at sites 1 inch beside the acupuncture points used in the acupuncture group;  The needles were connected to an electric stimulator but with zero frequency and amplitude. |  |  |  |
| 7 | (Dong et al., 2007) | Dong 2007 | RCT;  Single-center | China | PSD | **Stroke:**  Diagnostic criteria revised at the 4th National Conference on cerebrovascular disease (1995);  Stroke diagnosis by CT or MRI.  **Depression:**  CCMD-3;  DSM-IV. | **Inclusion:**  With first-onset stroke (hemorrhage or infarction in CT or MRI);  With depression (HAMD-/: >20; SDS score index >60%);  31-70 yr.  **Exclusion:**  With serious physical diseases or drug addiction;  Pregnant or lactating;  Taking SSRIs within 2 weeks;  Serious suicidal tendency;  Primary and secondary depression of non-stroke causes. | EA | 58.4±9.6 | 25/13 | 4 weeks | Once a day, 10 times as a course of treatment, a total of 3 courses of treatment. | **Acupoints:** GB5 through GB6, GV17 through GV18, GB15 through GB14, GB8 through GB7, GV24 through GV29;  **Insertion:** 40-50 mm;  **Current:** Continuous wave, 120-250 times/min;  **Retaining time:** min. EA for 30 min and retain the needle for 30 min. | 0d;  4wk (30d). | HAMD-/;  SDS;  Serum 5-HT. | / |
|  |  |  |  |  |  |  |  | MA | 59.21±7.56 | 23/13 | 4 weeks | Once a day, 10 times as a course of treatment, a total of 3 courses of treatment. | **Acupoints:** GV20, GV29, EX-HN1, SP6, PC6, LR3, HT7;  Manual manipulation;  **Retaining time:** 30 min. |  |  |  |
|  |  |  |  |  |  |  |  | Fluoxetine | 56.61±8.21 | 19/15 | 4 weeks | Sid. | Fluoxetine: 0-2w: 20 mg/d; 2w-4w: 80 mg/d; |  |  |  |
| 8 | (Duan, 2008) | Duan 2008 | RCT;  Single-center | China | Depression | CCMD-3;  Diagnostic efficacy standard of TCM diseases. | **Inclusion:**  HAMD-/: 20-35, somatic symptoms: ≥ 4;  18-60 yr.  **Exclusion:**  With schizophrenia, other mental disorders, organic diseases, severe brain organic disease, somatic disease,;  Pregnant, potentially pregnant, lactating;  Severe depression (HAMD-/: ≥35) or suicidal tendency;  Intolerance and allergy to Fluoxetine. | Fluoxetine | 39.47±11.20 | 8/17 | 6 weeks | Sid. | Fluoxetine: 20mg/d; | 0wk;  1wk;  2wk;  3wk;  4wk;  5wk;  6wk. | HAMD-/;  SDS;  TESS;  AEs. | 6.67% |
|  |  |  |  |  |  |  |  | EA | 40.42±10.71 | 9/16 | 6 weeks | Once a day, 5 times a week, 2 days off for the next week of treatment. | **Acupoints:** GV20, GV29; LR3, LI4, LR2, GB43, Anmian, HT7, PC6, SP6, ST36, KI3, KI6;  **Insertion:** transverse insertion in GV20 13-20mm; GV29 7-13mm;  **Current:** Continuous wave, 2Hz;  **Retaining time:** EA for 30-40 min, Retaining needle for 30 min. |  |  |  |
|  |  |  |  |  |  |  |  | Fluoxetine + EA | 38.17±11.31 | 8/17 | 6 weeks | **Fluoxetine:** Sid;  **EA:** once a day, 5 times a week, 2 days off for the next week of treatment. | Fluoxetine: same as the fluoxetine group;  EA: same as the EA group |  |  |  |
| 9 | (Duan et al., 2011) | Duan 2011 | RCT;  Single-center;  Blind method used (assessors) | China | Depression | ICD-10. | **Inclusion:**  First onset depression;  HAMD-24: 20-34, single episode depression;  18-50 yr.  **Exclusion:**  With schizophrenia, mental disorders, organic diseases, central nervous system diseases;  Pregnant, potentially pregnant, lactating;  Severe or relapsed depression (HAMD-24: ≥35) or suicidal tendency;  Intolerance and allergy to Fluoxetine. | Fluoxetine | 35±8 | 6/49 | 6 weeks | Sid. | Fluoxetine: 20mg/d, 6 weeks in total; | 0wk;  6wk. | HAMD-17;  MRI;  AEs. | 6.67% |
|  |  |  |  |  |  |  |  | EA+Fluoxetine |  |  | 6 weeks | **Fluoxetine:** Sid;  **EA:** once a day, 5 times a week, 2 days off for the next week of treatment. | Fluoxetine: same as the fluoxetine group;  **Acupoints:** GV20, GV29; EX-HN1, Anmian, SP6, KI3, KI6, GV24, GB13, GB20, CV12, ST25, ST34, ST40, ST36, ST44, HT7, PC6(selected according to Syndrome Differentiation);  **Insertion:** transverse insertion in GV20 13-20mm; GV29 7-13mm;  **Current:** continuous wave, 2Hz;  **Retaining time:** EA for 30-40 min, Retaining needle for 30 min. |  |  |  |
| 10 | (Han et al., 2019) | Han 2019 | RCT;  Single-center | China | First episode mild to moderate depression | DSM-V. | **Inclusion:**  First onset depression;  Mild to moderate depression (HAMD-/: 20-35);  18-60 yr;  Junior high school education or above.  **Exclusion:**  With other mental disorders, organic diseases, central nervous system diseases, addiction;  Severe depression with psychotic symptoms;  Suicidal tendency;  Seasonal depression;  Secondary depression;  Using other therapies for depression within 2 weeks. | MA | 39.0 (35.0, 46.5) statistical meaning unknown | 8/17 | 6 weeks | Every other day, 3 times a week. | **Acupoints:** same as the EA group;  **Insertion:** same as the EA group;  Manual manipulation: no manipulation methods;  **Retaining time:** 30 min. | 0wk;  6wk;  6m (followk-up) | HAMD-/;  SDS. | 10% |
|  |  |  |  |  |  |  |  | EA | 37.0 (32.0, 41.5) statistical meaning unknown | 8/17 | 6 weeks | Every other day, 3 times a week. | **Acupoints:** GV20, GV29, EX-HN1, EX-HN5, LI4;  **Insertion:** perpendicular, oblique or transverse insertion 15mm;  **Current:** continuous wave, 1 Hz;  **Retaining time:** 30 min. |  |  |  |
| 11 | (He et al., 2011) | He 2011 | RCT;  Single-center;  Blind method used (investigators, assessors, data collectors and statisticians) | China | Comorbidity of anxiety and depression (CAD) | CCMD-3. | **Inclusion:**  Diagnosis of anxiety and depression;  Hamilton Anxiety Scale (HAMA): > 14;  HAMD-/: > 17.  **Exclusion:**  Serious physical diseases, schizophrenia and brain organic diseases;  Other mental disorders;  Pregnancy or lactating. | rTMS | 40.3±13.0 | 13/32 | 10 days | Once a day, 10 times in total. | **rTMS:** 0-5d: 1Hz low frequency, 90% motor threshold; 5d-10d: 10Hz high frequency, 90% motion threshold. | 0d;  5d;  10d. | HAMD-/;  HAMA. | 23.5% |
|  |  |  |  |  |  |  |  | EA+rTMS | 35.4±9.1 | 13/27 | 10 days | Once a day, 10 times in total. | **rTMS:** same as the rTMS group;  **Acupoints:** GV20, GV29, GV24, PC6, HT7, LI4, SP6, EX-HN1, EX-HN18, LR3;  **Insertion:** /;  **Current:** Dilatational wave, 4/20Hz(10/15s);  **Retaining time:** 30 min. |  |  |  |
| 12 | (Li and Liu, 2007) | Li 2007 | RCT;  Single-center | China | Depression | CCMD-3. | **Inclusion:**  Diagnosis of depression;  HAMD-24: >20;  BDI: >5.  **Exclusion:** /. | EA | 49.19±13.46 | 15/17 | 6 weeks | Once a day. | **Acupoints:** GV20, GV29, EX-HN1, HT7, SP6, LR3, ST36, GB8, PC5, LI4, GB20, EX-HN22, GB40, CV12;  **Insertion:** /;  **Current:** /;  **Retaining time:** 30 min. | 0wk;  6wk. | HAMD-24;  Onset time;  Effective time. | / |
|  |  |  |  |  |  |  |  | Fluoxetine/Paroxetine | 47.00 ±13.08 | 10/14 | 6 weeks | Sid. | **Fluoxetine/paroxetine:** 20mg/d; |  |  |  |
| 13 | (Li et al., 2015) | Li 2015 | RCT;  Single-center | China | PSD | Diagnostic essentials of various cerebrovascular diseases revised at the fourth national cerebrovascular disease academic conference in 1996. | **Inclusion:**  First onset depression, course ≤ 3 months;  With stroke (CT or MRI);  HAMD-17: >18.  **Exclusion:**  Consciousness disorder, severe intellectual disorder, logopathy, brain organic disease, mental disorder, addition, serious physical diseases;  Received antidepressants before. | Fluoxetine | 66.42±6.25 | 5/5 | 8 weeks | Sid. | **Fluoxetine:** 20mg/d; | 0wk;  2wk;  4wk;  8wk. | HAMD-17;  SPECT. | 0% |
|  |  |  |  |  |  |  |  | EA | 62.56±6.85 | 4/7 | 8 weeks | Once a day, 5 days a week. | **Acupoints:** LR3, LI4;  **Insertion:** 15mm;  **Current:** Dilatational wave, 2Hz;  **Retaining time:** 20 min. |  |  |  |
| 14 | (Li et al., 2020) | Li 2020 | RCT;  Single-center;  Blind method used (assessors and statisticians) | China | Moderate depression | DSM-IV. | **Inclusion:**  Moderate to severe depression (HAMD-/: 20-35);  18-70 yr.  **Exclusion:**  With severe diseases, mental disorder, brain diseases, addiction;  Concurrently participate in other depression-related trials;  Pregnant or lactating;  Taken acupuncture within 6 months. | Antidepressants | 38.75±11.45 | 11/19 | 8 weeks | **Antidepressants:** /; | Continue to take the routine antidepressants in the same amount and frequency as before. | 0wk;  4wk;  8wk;  12wk (followk-up). | HAMD-24;  Urinary metabolites. | 11.67% |
|  |  |  |  |  |  |  |  | EA+antidepressants | 40.3±10.99 | 11/19 | 8 weeks | **Antidepressants:** /;  **EA:** once every other day, 3 times a week. | Continue to take the routine antidepressants in the same amount and frequency as before;  **Acupoints:** GV20, GV29, GV24, HT7, PC6, ST36, SP6;  **Insertion:** /;  **Current:** continuous wave, 10Hz (GV20, GV29);  **Retaining time:** /. |  |  |  |
| 15 | (Lu et al., 2018) | Lu 2018 | RCT;  Single-center | China | Post schizophrenic depression | ICD-10. | **Inclusion:**  Diagnosis of depression;  HAMD-17: >17;  Junior high school degree or above;  18-45 yr.  **Exclusion:**  Taking or in washout phase of antidepressants;  Taking antiarrhythmic, hypoglycemic or tryptophan drugs;  Pregnant or lactating;  With serious physical diseases, infectious diseases, systemic diseases, brain organic diseases, psychotropic drugs abuse;  Suicidal tendency (NGASR: <11). | EA+sertraline | 29.6±11.2 | 16/14 | 6 weeks | **Sertraline:** Sid;  **EA:** once every other day, 3 times a week. | **Sertraline:** same as the sertraline group;  **Acupoints:** GV20, GV29;  **Insertion:** 13-20 mm, no lifting and thrusting;  **Current:** GV20 (positive electrode), GV29 (negative electrode), dilatational wave, 2/15Hz;  **Retaining time:** 30 min. | 0wk;  1wk;  2wk;  4wk;  6wk. | GAS;  HAMD-17;  AEs. | 8.33% |
|  |  |  |  |  |  |  |  | Sertraline | 29.2±10.5 | 17/13 | 6 weeks | Sid. | **Sertraline:** 0-1w: 50mg/d; 1w-6w: 100mg/d; |  |  |  |
| 16 | (Luo et al., 1985) | Luo 1985 | RCT;  Single-center | China | Depression | DSM-III. | **Inclusion:**  Diagnosis of depression;  HAMD-24: ≥20.  **Exclusion:**  /. | EA | 39 | 15/12 | 5 weeks | Once a day except Sunday. | **Acupoints:** GV20, GV29;  **Insertion:** obliquely insertion, 0.8 fen;  **Current:** 80-90 beats/min;  **Retaining time:** 60 min. | 0wk;  1wk;  2wk;  3wk;  4wk;  5wk. | HAMD-24;  CGI;  SERS (Asberg). | 0% |
|  |  |  |  |  |  |  |  | Amitriptyline | 35 | 6/14 | 5 weeks | Tid. | **Amitriptyline:** 0-1w: 25mg, 3 times/week; 2-5w: 100-200mg/d (142 on average). |  |  |  |
| 17 | (Luo et al., 1988) | Luo 1988 | RCT;  Multi-center;  Single-blind (medication placebo) | China | Depression | National diagnostic criteria for manic depressive disorder;  CCMD;  ICD-9. | **Inclusion:**  Diagnosis of depression;  HAMD-/: >20.  **Exclusion:** /. | EA+Placebo | 32 (min-max: 17-64) | 109 /132 | 6 weeks | **Placebo:** /;  **EA:** once a day. | **Placebo:** 25mg/d;  **Acupoints:** GV20, GV29;  **Insertion:** /;  **Current:** 6V, 8-9ma, 80-100 times/min;  **Retaining time:** 60 min. | 0wk;  1wk;  2wk;  3wk;  4wk;  5wk;  6wk. | HAMD-/;  CGI;  SERS (Asberg);  AEs;  Biochemical test;  Electrophysiological examination. | 6.50% |
|  |  |  |  |  |  |  |  | Amitriptyline |  |  | 6 weeks | /. | **Amitriptyline:** begin in 25mg, increase 25-50mg/d, till 150mg. |  |  |  |
| 18 | (Luo et al., 1995) | Luo 1995 | RCT;  Multi-center | China | Depressive psychosis | Criteria presented at the Huangshan Symposium on manic-depression;  Handbook of epidemiological investigation mental illness in China. | **Inclusion:**  Diagnosis of depression;  HAMD-24: ＞ 20.  **Exclusion:**  Taken medication for at least 1 week. | EA | 36 | 32/22 | 5 weeks | Once a day. | **Acupoints:** GV20, GV29;  **Insertion:** 8 fen;  **Current:** 3-5ma, 2Hz, 3-5V;  **Retaining time:** 45-60 min. | 0wk;  1wk;  2wk;  3wk;  4wk;  5wk;  6wk. | HAMD-24;  CGI;  SERS (Asberg). | / |
|  |  |  |  |  |  |  |  | Amitriptyline |  | 19/28 | 5 weeks | Tid. | **Amitriptyline:** 0-1w: 25mg; 1-5w: adjust according effects and side effects, up to 50mg; |  |  |  |
| 19 | (Ma et al., 2011) | Ma 2011 | Parallel RCT;  Multi-center;  Blind method used (statisticians) | China | Mild or moderate depression | ICD-10. | **Inclusion:**  First onset of depression and without systematic treatment;  HAMD-17: ≥17;  18-60 yr.  **Exclusion:**  Participated in other clinical trials within 4 weeks;  Taking antidepressants or in washout phase;  Pregnant or lactating;  With other serious systemic diseases, brain diseases, other mental disorder;  Severe depression;  Suicidal tendency. | Paroxetine | 40.52±14.21 | 19/10 | 6 weeks | Sid. | **Paroxetine:** 0d-2d: 10mg/d, once a day; 2d-6w: 20mg/d, once a day, oral after breakfast; | 0wk;  1wk;  2wk;  4wk;  6wk. | HAMD-17;  SERS;  CGI;  AEs. | 3.64% |
|  |  |  |  |  |  |  |  | EA | 46.27±13.13 | 17/9 | 6 weeks | Once every other day, 3 times a week. | **Acupoints:** GV20, GV29, GV16, GB20, SP6, PC6, CV14;  **Insertion:** /;  **Current:** 2/15Hz, dilatational wave (GV20, GV29, bilateral GB20);  **Retaining time:** 30 min (manipulation at 15min). |  |  |  |
| 20 | (Qu, 2015) | Qu 2015 | RCT;  Multi-center;  Blind method used (statisticians) | China | Depression | ICD-10. | **Inclusion:**  Moderate or severe depression (HAMD-17: >17);  18-60 yr.  **Exclusion:**  Secondary depression;  Participated in other clinical trials within 4 weeks;  Taking antidepressants or in washout phase;  Taking antiarrhythmic, hypoglycemic or tryptophan drugs;  Pregnancy or lactating;  With severe cardiovascular, liver, kidney or hematopoietic system, brain disease;  Suicidal tendency. | Paroxetine | 35.58±10.62 | 31/34 | 6 weeks | Sid. | **Paroxetine:** 0-1d: 10mg/d; 3d-6w: 20mg/d. | 0wk;  1wk;  2wk;  4wk;  6wk. | HAMD-17;  SDS;  SERS;  AEs. | 2.05% |
|  |  |  |  |  |  |  |  | EA+paroxetine | 34.03±10.60 | 28/34 | 6 weeks | **Paroxetine:** Sid;  **EA:** once every other day, 3 times a week. | **Paroxetine:** same as paroxetine group;  **EA:** same as EA group; |  |  |  |
|  |  |  |  |  |  |  |  | EA | 33.2±9.0  36.58±10.9 | 23/41 | 6 weeks | Once every other day, 3 times a week. | **Acupoints:** GV20, GV29, GV16, GV14, GB20, PC6, SP6;  **Insertion:** 0.5-1.2 cun;  **Current:** dilatational wave, 2/15Hz;  **Retaining time:** 30 min (manipulation at 15min). |  |  |  |
| 21 | (Song et al., 2007) | Song 2007 | RCT;  Single-center;  Single-blind (patients and assessors) | China | Depression | DSM-IV. | **Inclusion:**  Diagnosis of depression;  HAMD-24: ≥20.  **Exclusion:**  Suicidal tendency;  With psychotic symptoms. | Sham acupoints acupuncture+fluoxetine | 33.9 ±12.4 | 81(Sex not covered in the original text) | 6 weeks | **Fluoxetine:** Sid;  **Sham acupoints acupuncture:** every weekend. | **Fluoxetine:** 20mg/d;  **Sham acupoints acupuncture:** same as the sham acupoints acupuncture+placebo group. | 0wk;  6wk. | HAMD-24;  Serum G protein. | / |
|  |  |  |  |  |  |  |  | EA+placebo | 30.8±10.9 |  | 6 weeks | **Placebo:** Sid;  **EA:** Every weekday. | **Placebo:** 20 mg/d;  **Acupoints:** GV20, GV29;  **Insertion:** /;  **Current:** 10-40 mA;  **Retaining time:** 45 min. |  |  |  |
|  |  |  |  |  |  |  |  | Sham acupoints acupuncture+placebo | 30.5±12.0 |  | 6 weeks | **Placebo:** Sid;  **Sham acupoints acupuncture:** Every weekday. | **Placebo:** 20mg/d;  **Sham acupoints acupuncture:** a needle punctured under the skin at the side of 1 cm far from away from GV20 and GV29 points, no current. |  |  |  |
| 22 | (Song et al., 2009) | Song 2009 | RCT;  Single-center;  Single-blind (patients and assessors) | China | Depression | DSM-IV. | **Inclusion:**  Diagnosis of depression;  HAMD-24 (21 in the article): ≥20.  **Exclusion:**  With other mental disorders, addiction, severe smoking;  Abnormal in physical examinations, routine laboratory tests and ECG;  Physical disorders that would necessitate medical interventions;  With injury, acute diseases or surgery within 4 weeks;  Taken fluoxetine within 4 weeks;  Taken other antidepressants within 3 weeks;  Taken neuroleptic drugs and drug that may affect the immune system within 3 years;  Receiving acupuncture treatment;  With severe metabolic or endocrine disorder;  Suicidal tendency;  Pregnancy or lactating within 6 months. | EA+placebo | 30±11 | 13/19 | 6 weeks | **Placebo:** Sid;  **EA:** 3 times a week. | **Placebo:** 20mg/d;  **Acupoints:** GV20, GV29;  **Insertion:** /;  **Current:** anti-depression sequence of waves;  **Retaining time:** 45 min. | 0wk;  1wk;  2wk;  3wk;  4wk;  5wk;  6wk. | Serum cytokine;  HAMD-24 (21 in the article);  CGI. | 12.63% |
|  |  |  |  |  |  |  |  | Sham acupoints acupuncture+fluoxetine | 34±13 | 13/18 | 6 weeks | **Fluoxetine:** Sid;  **Sham acupoints acupuncture:** 3 times a week. | **Fluoxetine:** 20mg/d;  **Sham acupuncture:** same as the sham acupoints acupuncture+placebo group. |  |  |  |
|  |  |  |  |  |  |  |  | Sham acupoints acupuncture+placebo | 30±12 | 13/19 | 6 weeks | **Placebo:** Sid;  **Sham acupoints acupuncture:** 3 times a week. | **Placebo:** 20mg/d;  **Sham** **acupoints:** 1cm beside GV20 and GV29;  **Current:** anti-depression sequence of waves;  **Retaining time:** 45 min. |  |  |  |
| 23 | (Sun et al., 2010) | Sun 2010 | RCT;  Multi-center | China | Depression | CCMD-3;  DSM-Ⅳ. | **Inclusion:**  Diagnosis of depression;  HAMD-24: ≥17;  18-70 yr;  Fits the syndrome diagnose of TCM.  **Exclusion:**  With severe physical diseases, endocrine, immune and nervous system diseases‚ chronic infection, other mental diseases, addiction;  Taken immunosuppressant or immune enhancer within 6 months;  Taken electric shock treatment therapy;  Taken antipsychotics, antidepressants within 2 weeks;  Abnormal in routine laboratory tests and ECG;  With major life events within 1 year;  Pregnancy or lactating. | EA1 | 35.9±14.5 | 13/15 | 6 weeks | Once a day, 5 days a week, 2 days off on weekends. | **Acupoints:** GV20, ST36;  **Insertion:** /;  **Current:** 3Hz, continuous wave;  **Retaining time:** 30 min. | 0wk;  2wk;  4wk;  6wk. | HAMD-24;  Serum IL-1β、IL-6、TNF-α. | 6.67% |
|  |  |  |  |  |  |  |  | EA2 | 41.1±11.5 | 5/23 | 6 weeks | Once a day, 5 days a week, 2 days off on weekends. | **Acupoints:** SP6, PC6, LR3, HT7;  **Insertion depth, current, retaining time:** same as the EA1 group. |  |  |  |
|  |  |  |  |  |  |  |  | Fluoxetine | 39.1±13.2 | 3/25 | 6 weeks | Sid. | **Fluoxetine:** 20mg/d. |  |  |  |
| 24 | (Sun et al., 2013) | Sun 2013 | RCT;  Single-center;  Blind method used (assessors of GDNF) | China | Depression | DSM-Ⅳ. | **Inclusion:**  Diagnosis of depression;  18-70 yr;  HAMD-24: ≥20.  **Exclusion:**  With other mental diseases, mental retardation, addiction, severe physical diseases, brain disease and obesity;  With infection, autoimmune diseases within 6 months;  Taken electric shock treatment therapy, immunosuppressant or immune enhancer within 6 months;  Pregnancy or lactating. | EA1 | 43.10±13.86 | 8/12 | 6 weeks | Once a day, 5 times a week. | **Acupoints:** GV20, ST36;  **Insertion:** 0.25-0.4cm;  **Current:** 3Hz, continuous wave;  **Retaining time:** 30 min. | 0wk;  2wk;  4wk;  6wk. | HAMD-24;  Serum GDNF. | 18.67% |
|  |  |  |  |  |  |  |  | EA2 | 42.56±10.70 | 3/13 | 6 weeks | Once a day, 5 times a week. | **Acupoints:** LR3, SP6, PC6, HT7;  **Insertion:** 0.25-0.4cm;  **Current:** 3Hz, continuous wave;  **Retaining time:** 30 min. |  |  |  |
|  |  |  |  |  |  |  |  | Fluoxetine | 40.72±12.80 | 3/22 | 6 weeks | Sid. | Fluoxetine: 20mg/d. |  |  |  |
| 25 | (Wang et al., 2013) | Wang 2013 | RCT;  Single-center;  Non-blind | China | Depression | DSM-IV. | **Inclusion:**  Diagnosed of depression;  18-60 yr.  **Exclusion:**  Suicidal tendency;  Pregnancy or lactating;  With other mental disorders or substance abuse;  Taken antidepressants or other psychotropic medications within 2 weeks. | EA | 48.10±13.40 | 7/17 | 24 weeks | 3 times a week. | **Acupoints:**GV20, GV29, EX-HN1, PC6, HT7, SP6;  **Insertion:** transverse, perpendicular insertion 0.5-1cm;  **Current:** 125 ms, intermittent pulses, 40 Hz (EX-HN1, PC6);  **Retaining time:** 20min. | 0wk;  24wk. | MMPI;  SDS;  SAS;  MADRS;  AEs. | 20% |
|  |  |  |  |  |  |  |  | Paroxetine | 47.10±10.60 | 8/16 | 24 weeks | Sid. | **Paroxetine:** 0-2w: 20mg/d after breakfast every day; 2-24w: adjust the dose in 10mg increments, and the maximum 60mg/d; |  |  |  |
| 26 | (Wang et al., 2014) | Wang 2014 | Parallel RCT;  Multi-center | China | Mild or moderate depression | ICD-10. | **Inclusion:**  First onset of depression;  HAMD-17: ≥ 17;  18-60 yr.  **Exclusion:**  With systematic treatment;  Bipolar depression;  Severe depression;  Participated in other clinical trials within 4 weeks;  Taking or in washout phase of antidepressants;  Pregnancy and lactating;  With severe systemic diseases, brain diseases;  Suicidal tendency. | Paroxetine | 48±9 | 6/11 | 6 weeks | Sid. | **Paroxetine:** 0-2d: 10mg/d; 2d-6w: 20mg/d; | 0wk;  1wk;  2wk;  4wk;  6wk;  10wk (followk-up). | HAMD-17;  SERS;  WHOQOL-Bref;  AEs. | 12.50% |
|  |  |  |  |  |  |  |  | MA+paroxetine | 45±12 | 10/22 | 6 weeks | **Paroxetine:** Sid;  **MA:** once every other day, 3 times a week. | **Paroxetine:** same as the paroxetine group;  **Acupoints:** GV20, GV29, GV16, GB20, SP6, PC6, CV14;  **Insertion:** /;  Manual manipulation;  **Retaining time:** 30 min (manipulation at 15min). |  |  |  |
|  |  |  |  |  |  |  |  | EA+paroxetine | 47±11 | 3/20 | 6 weeks | **Paroxetine:** Sid;  **EA:** once every other day, 3 times a week. | **Paroxetine:** same as the paroxetine group;  **Acupoints:** GV20, GV29, GV16, GB20, SP6, PC6, CV14;  **Insertion:** /;  **Current:** dilatational wave 2/15Hz (GV20, GV29, bilateral GB20);  **Retaining time:** 30 min (manipulation at 15min). |  |  |  |
| 27 | (Xu and Wang, 2011) | Xu 2011 | RCT;  Single-center (part of multi-center) | China | Depression | CCMD-3. | **Inclusion:**  Diagnosis of depression;  HAMD-17: ≥17;  18-60 yr.  **Exclusion:**  Taken SSRI within 4 weeks or stopped other antidepressants within 1 week; with serious physical diseases, immune dysfunction, brain organic diseases, addiction and allergic constitution;  Taking hormone drugs, nerve blockers and immune modulators within 1 month. | SSRIs | 47.42±8.89 | 11/19 | 6 weeks | / | **SSRIs (fluoxetine/paroxetine/sertraline/citalopram/fluvoxamine):** routine dose. | 0wk;  1wk;  2wk;  4wk;  6wk | HAMD-17. | 0% |
|  |  |  |  |  |  |  |  | MA+SSRIs | 48.01±8.16 | 10/15 | 6 weeks | **SSRIs:** /;  **MA:** once every other day, 3 times a week. | **SSRIs:** same as the SSRIs group;  **Acupoints:** GV20, GV29, GV24, GB20, CV14, GV11, GV9, BL15, ST36, KI3, BL18, BL19, HT7;  **Insertion:** /;  Manual manipulation;  **Retaining time:** 30 min. |  |  |  |
|  |  |  |  |  |  |  |  | EA+SSRIs | 47.54±8.03 | 7/13 | 6 weeks | **SSRIs:** /;  **EA:** once every other day, 3 times a week. | SSRIs: same as the SSRIs group;  **Acupoints:** GV20, GV29, GB20;  **Insertion:** /;  **Current:** dilatational wave 2/15Hz;  **Retaining time:** 30 min. |  |  |  |
| 28 | (Yan et al., 2004) | Yan 2004 | RCT;  Single-center;  Blind method used (assessors) | China | Depression | CCMD. | **Inclusion:**  Diagnosis of depression;  HAMD-/: >20.  **Exclusion:** /. | EA | 38±5 | 10/9 | 30 times (possibly 6 weeks) | Once a day. | **Acupoints:** GV20, GV29;  **Insertion:** obliquely insertion 1 cun;  **Current:** 80-90 times/min;  **Retaining time:** 30min. | 0wk;  1wk;  2wk;  3wk;  4wk;  5wk;  6wk. | HAMD-/;  CGIS. | 0% |
|  |  |  |  |  |  |  |  | Amitriptyline | 36±8 | 3/8 | 6 weeks | Tid. | **Amitriptyline:** 0-1w: 250 mg/d; 1-6w: increase according to the symptoms and side effects, with an average 130 mg/d; |  |  |  |
| 29 | (Yang et al., 1994) | Yang 1994 | RCT;  Single-center | China | Mental depression | Clinical criteria for diagnosis of manic depression. | **Inclusion:**  Diagnosis of depression;  HAMD-24: >25.  **Exclusion:** /. | EA | 22-57 | 17/24 | 6 weeks | 6 times a week, followed by 1 day off; | **Acupoints:** GV24, GV20, GV14, GV12, CV17, CV14, GB20, PC6;  **Insertion:** /;  **Current:** 80-100 times/s;  **Retaining time:** / min. | 0wk;  1wk;  2wk;  3wk;  4wk;  5wk;  6wk. | HAMD-24;  EEG. | 0% |
|  |  |  |  |  |  |  |  | Amitriptyline |  |  | 6 weeks | /. | **Amitriptyline:** 0-1d: 25mg; 1-2w: increase 25-50mg/d to 150 mg/d; 2w-6w: according to the dose of side effects and pathological changes, the daily minimum dose is changed from 150mg to 300mg; |  |  |  |
| 30 | (Yeung et al., 2011) | Yeung 2011 | Parallel RCT;  Single-center;  Single-blind (patients and assessors) | Hongkong | Depression | DSM-IV;  With insomnia complaint. | **Inclusion:**  Previous diagnosis of depression;  HAMD-17≤18;  Taken the same antidepressants at fixed doses for at least 12 weeks before 0w;  With Insomnia complaints;  18-65 yr.  **Exclusion:**  With symptoms suggestive of specific sleep disorders;  With apnea-hypopnea index ≥ 10 or a periodic limb movement disorder index with arousal ≥ 15 (according to actigraphy measures);  Suicidal tendency;  Previous diagnosis of schizophrenia, other mental disorders, bipolar disorder or addiction;  Pregnant, lactating or fertile;  With severe physical diseases, heart valve defect or hemorrhagic disease or taking anticoagulant drugs;  Infection or abscess near the intervention acupoint;  Taking Chinese herbal medicine or over-the-counter medicine for insomnia. | EA+antidepressants | 47.5±8.5 | 6/20 | 3 weeks | **Antidepressants:** /;  **EA:** 3 times a week. | Continue the same type and dosage of antidepressants throughout the study period;  **Acupoints:** GV29, GV20, EX-HN1, HT7, EX-HN22;  **Insertion:** /;  **Current:** /;  **Retaining time:** /min. | 0wk;  1wk;  4wk (followk-up). | ISI;  PSQI;  Sleep diary;  Actigraphy measures;  HAMD-17;  AEs. | 9% |
|  |  |  |  |  |  |  |  | Sham acupoint acupuncture+antidepressants | 46.7±9.7 | 7/19 | 3 weeks | **Antidepressants:** /;  **Sham acupoints acupuncture:** 3 times a week. | Continue the same type and dosage of antidepressants throughout the study period;  **Sham** **acupoints:** Bilateral “deltoids” (between LI14 and acromion line), “forearms” (1 inch outside the midpoint of HE3 and HE7), “upper arms” (1 inch outside Lu3), “lower legs” (0.5 inch on the back of GB39). |  |  |  |
|  |  |  |  |  |  |  |  | Non-invasive acupuncture +antidepressants | 50.1±9.1 | 3/23 | 3 weeks | **Antidepressants:** /;  **Non-invasive acupuncture:** 3 times a week. | Continue the same type and dosage of antidepressants throughout the study period;  Place the placebo needle 1 inch next to the acupoint to avoid acupoint pressing;  Electrode current frequency and amplitude are 0. |  |  |  |
| 31 | (Yin et al., 2020) | Yin 2020 | RCT;  Single-center;  Single-blind (patients and assessors) | China | Depression with insomnia | DSM-IV. | **Inclusion:**  Mild to moderate depression (HAMD-17: 20-35);  With Insomnia complaints at the first screening;  PSQI: >7;  Poor sleep quality (Sleep efficiency < 85%, recorded by actigraphy);  18-70 yr.  **Exclusion:**  With secondary depressive disorders, addiction, severe cardiovascular, liver, kidney or hematopoietic system disease;  Depressive episode of bipolar disorder;  Had infections, ulcers and scars at the intervention acupoints;  Taken acupuncture treatment within 1 year;  Pregnancy or lactating. | EA+antidepressants | 47.30±14.89 | 11/19 | 8 weeks | **Antidepressants:** /;  **EA:** 3 times a week. | Continue to take the routine antidepressants in the same amount and frequency as before;  **Acupoints:** GV20, GV24, GV29, EX-HN22, HT7, SP6, PC6;  **Insertion:** /;  **Current:** 0.175ms, 30Hz, 0.1-1ma, continuous wave, square pulse (GV20, GV29);  **Retaining time:** 30 min. | 0wk;  4wk;  8wk;  12wk (followk-up). | PSQI;  SE;  TST;  SA;  HAMD-17;  SDS;  HAMA;  AEs. | 14.44% |
|  |  |  |  |  |  |  |  | Sham acupoint acupuncture+antidepressants | 49.80±15.13 | 10/20 | 8 weeks | **Antidepressants:** /;  **Sham acupoints acupuncture:** 3 times a week. | Continue to take the routine antidepressants in the same amount and frequency as before;  **Sham** **acupoints:** ST6, SJ5, SJ4, GB39 taking at 1cm outside; SI18 upper and outer acupoints; Bilateral acupoints between SJ16 and SI17; 1 cm away from Yang Meridian Acupoints, opposite to Yin meridian acupoints selected by real Electroacupuncture; Other fake acupoints are far away from the governor vessel and its branches;  Electrodes connected at 1cm from the upper and outer sides of ST6 on both sides with the switch but 0 current. |  |  |  |
|  |  |  |  |  |  |  |  | Non-invasive acupuncture+antidepressants | 46.77±15.57 | 11/19 | 8 weeks | **Antidepressants:** /;  **Non-invasive acupuncture:** 3 times a week. | Continue to take the routine antidepressants in the same amount and frequency as before;  Use blunt needle tip contacts the skin;  A pair of electrodes are connected at 1 cm from the upper and outer sides of ST6 on both sides, with 0 current. |  |  |  |
| 32 | (Zhang et al., 2007) | Zhang 2007 | RCT;  Single-center | China | Depression | CCMD-3. | **Inclusion:**  Diagnosis of depression;  HAMD-17: >18;  18-65 yr.  **Exclusion:**  Suicidal tendency;  With severe somatopathy, addiction;  Pregnancy or lactating. | Paroxetine | 37.1±10.2 | 9/11 | 6 weeks | Sid. | **Paroxetine:** 10-40mg/d; | 0wk;  1wk;  2wk;  4wk;  6wk. | HAMD-17;  TESS;  Routine blood, urine test;  ECG. | 0% |
|  |  |  |  |  |  |  |  | EA+paroxetine | 36.6+9.7 | 12/10 | 6 weeks | **Paroxetine:** Sid;  **EA:** once a day, 6 times a week. | **Paroxetine:** same as the Paroxetine group;  **Acupoints:** GV20, GV29, PC6, SJ5, HT7, LI4, LR36, ST36, ST40, SP6, LU9; SP10, CV17, CV22, HT7, anmian;  **Insertion:** /;  **Current:** dilatational wave, 2Hz, 6V;  **Retaining time:** 30 min. |  |  |  |
| 33 | (Zhang et al., 2020) | Zhang 2020 | RCT;  Single-center | China | PCOS with mild anxiety/depression | Rotterdam PCOS diagnostic criteria;  With mild anxiety/depression. | **Inclusion:**  Diagnosis of PCOS;  Mild anxiety/depression or above (SAS/SDS);  18-40 yr.  **Exclusion:**  With hyperandrogenemia caused by other reasons;  With severe diseases, such as kidney disease, liver dysfunction, autoimmune disease or cancer;  Taken cortisol, hypoglycemic treatment, antidepressants, hormonal contraceptives, hormonal ovulation stimulating drugs within 3 months; Taken medroxyprogesterone or similar drugs within 6 months;  With other mental diseases;  Pregnancy or lactating;  Taken acupuncture treatment within 2 months;  Needle fainting. | Lifestyle intervention | 28±3 | 0/40 | 4 months | 1 month is a course of treatment, a total of 4 courses of treatment. | **Dietary guidance:** avoid high-fat, fried food, drinks, food intake of 500-1000 kcal/d;  **Exercise guidance:** aerobic exercise for at least 30-40min/d, with exercise speed more than 6 km/h, more than 10min each exercise. | 0m;  4m (16wk). | BMI;  SAS;  SDS;  PCOSQ;  Hairiness score;  Serum sex hormone;  AEs. | 13.04% |
|  |  |  |  |  |  |  |  | EA+Lifestyle intervention | 29±2 |  | 4 months | **Lifestyle intervention:** at least 3 times a day; 1 month is a course of treatment, a total of 4 courses of treatment;  **EA:** once every other day, 3 times a week. | **Acupoints:** GV20, ST36, LI4, SP6, HT7, CV12, CV4, ST29, ST32, ST34;  **Insertion:** 15-40mm;  **Current:** continuous wave, 2Hz (CV12, CV4, ST29, ST32, ST34);  **Retaining time:** 30 min. |  |  |  |
| 34 | (Zhao et al., 2019) | Zhao 2019 | RCT;  Multi-center;  Single-blind (assessors) | China | Depression | ICD-10. | **Inclusion:**  First onset of moderate to severe depression episode (HAMD-17: ≥17);  18-60 yr.  **Exclusion:**  With bipolar depression, brain diseases, severe diseases;  Had participated in other clinical trials within 4 weeks;  Taking or in washout phase of antidepressants;  Pregnancy or lactating;  Suicidal tendency. | SSRIs | 41.76±12.85 | 57/99 | 6 weeks | Sid. | SSRIs(paroxetine/fluoxetine/sertraline/fluvoxamine/citalopram/escitalopram);  **Paroxetine:** 0-2d: 10mg/d; 3d: 20mg/d; 3d-6w: ;  Other SSRIs: Individualized to accommodate changes in symptom severity. | 0wk;  1wk;  2wk;  4wk;  6wk;  10wk (followk-up). | HAMD-17;  SDS;  CGI;  SERS;  AEs. | 20% |
|  |  |  |  |  |  |  |  | MA+SSRIs | 41.42±12.53 | 56/105 | 6 weeks | **SSRIs:** Sid;  **MA:** 3 times a week. | SSRIs: same as the SSRIs group;  **Acupoints:** GV20, GV29, GV16, GB20, GV14, PC6, SP6;  **Insertion:** perpendicular, transverse insertion, 0.5-1.2cun;  Manual manipulation;  **Retaining time:** 30 min (manipulation at 15min). |  |  |  |
|  |  |  |  |  |  |  |  | EA+SSRIs | 41.18±12.00 | 52/108 | 6 weeks | **SSRIs:** Sid;  **EA:** 3 times a week. | SSRIs: same as the SSRIs group;  **Acupoints:** GV20, GV29, GV16, GB20, GV14, PC6, SP6;  **Insertion:** perpendicular, transverse insertion, 0.5-1.2cun;  **Current:** dilatational wave, 2/15Hz (GV20, GV29, GB20);  **Retaining time:** 30 min (manipulation at 15min). |  |  |  |

/, Not covered in the original text; 5-HT, 5-hydroxytryptamine; AE, Adverse event; BI, Barthel Daily Living Index; BMI, Body Mass Index; CCMD, Chinese Classification and Diagnostic Criteria of Mental Disorders; CGI, Clinical Global Impression Scale; CGIS, Clinical Global Impressions Scale; CTRS, Credibility of Treatment Rating Scale; DSM, Diagnostic and Statistical Manual of Mental Disorders; EA, electroacupuncture; ECG, electrocardiograph; EEG, electroencephalogram; EPDS, Edinburgh Postpartum Depression Scale; ESS, Epworth Sleepiness Scale; GAS, Global Assessment Scale; GDNF, Glial Cell-Derived Neurotrophic Factor; HADS, Hospital Anxiety and Depression Scale; HAMA, Hamilton Anxiety Rating Scale; HAMD, Hamilton Depression Rating the Scale; ICD, International Classification of Diseases; NGASR, Nurses’ Global Assessment of Suicide Risk; MA, manual acupuncture; MADRS, Montgomery-Asberg Depression Rating Scale; MFI, Multidimensional Fatigue Inventory; MMPI, Minnesota Multiphasic Personality Inventory; NIHSS, National Institutes of Health Stroke Scale; PCOS, Polycystic ovary syndrome; PSD, Post stroke depression; PCOSQ, Polycystic Ovary Syndrome Quality of Life scale; PSQI, Pittsburgh Sleep Quality Index; RCTs, randomized controlled trials; SA, Sleep awake times; SAS, Self-Rating Anxiety Scale; SCL-90, Symptom Checklist-90; SDS, Self-Rating Depression Scale; SDSS, Social Disability Screening Schedule; SE, Sleep efficiency; SERS, Side Effects Rating Scale; SF-36, 36-item Short Form Health Survey; SSI, Somatic Symptom Inventory; TCM, Traditional Chinese Medicine; TESS, Treatment Emergent Symptoms Scale; TST, Total sleep time; WHOQOL-BREF, World Health Organization Quality of Life.

**Supplementary Table 2.** EA compared to MA for depression

| **Certainty assessment** | | | | | | | **№ of patients** | | **Effect** | | **Certainty** | **Importance** |
| --- | --- | --- | --- | --- | --- | --- | --- | --- | --- | --- | --- | --- |
| **№ of studies** | **Study design** | **Risk of bias** | **Inconsistency** | **Indirectness** | **Imprecision** | **Other considerations** | **EA** | **MA** | **Relative (95% CI)** | **Absolute (95% CI)** |  |  |
| **EA vs. MA at the end of treatment in severity of depression** | | | | | | | | | | | | |
| 3 | randomised trials | not serious | serious^a^ | not serious | serious^b^ | publication bias strongly suspected^c^ | 77 | 81 | - | MD **2.93**  (-1.96 to 7.82) | ⨁◯◯◯ Very low | Important |

CI: confidence interval; MD: mean difference

a. Considerable heterogeneity (I^2^>75%)

b. Total event number less than 400

c. All studies are from the same region/country

**Supplementary Table 3.** EA+antidepressants compared to MA+antidepressants for depression

| **Certainty assessment** | | | | | | | **№ of patients** | | **Effect** | | **Certainty** | **Importance** |
| --- | --- | --- | --- | --- | --- | --- | --- | --- | --- | --- | --- | --- |
| **№ of studies** | **Study design** | **Risk of bias** | **Inconsistency** | **Indirectness** | **Imprecision** | **Other considerations** | **EA+antidepressants** | **MA+antidepressants** | **Relative (95% CI)** | **Absolute (95% CI)** |  |  |
| **EA+antidepressants vs. MA+antidepressants in severity of depression at the end of treatment** | | | | | | | | | | | | |
| 4 | randomised trials | not serious | not serious | not serious | not serious | publication bias strongly suspected^a^ | 240 | 224 | - | MD **0.18**  (-0.11 to 0.46) | ⨁⨁⨁◯ Moderate | Not important |
| **EA+antidepressants vs. MA+antidepressants in severity of depression at week 2 during treatment** | | | | | | | | | | | | |
| 4 | randomised trials | not serious | not serious | not serious | serious^b^ | publication bias strongly suspected^a^ | 102 | 91 | - | MD **0.13**  (-0.22 to 0.47) | ⨁⨁◯◯ Low | Important |
| **EA+antidepressants vs. MA+antidepressants in severity of depression at week 4 during treatment** | | | | | | | | | | | | |
| 3 | randomised trials | not serious | not serious | not serious | serious^b^ | publication bias strongly suspected^a^ | 83 | 71 | - | MD **0.01**  (-0.3 to 0.32) | ⨁⨁◯◯ Low | Important |
| **EA+antidepressants vs. MA+antidepressants in severity of depression at week 6 during treatment** | | | | | | | | | | | | |
| 4 | randomised trials | not serious | not serious | not serious | not serious | publication bias strongly suspected^a^ | 240 | 224 | - | MD **0.18**  (-0.11 to 0.46) | ⨁⨁⨁◯ Moderate | Important |
| **EA+antidepressants vs. MA+antidepressants in adverse events** | | | | | | | | | | | | |
| 3 | randomised trials | not serious | not serious | not serious | not serious | publication bias strongly suspected^a^ | 10/228 (4.4%) | 15/218 (6.9%) | **RR 0.65** (0.30 to 1.37) | **-** | ⨁⨁⨁◯ Moderate | Important |

CI: confidence interval; MD: mean difference; RR: risk ratio

a. All studies are from the same region/country

b. Total event number less than 400

**Supplementary Table 4.** EA compared to control for depression

| **Certainty assessment** | | | | | | | **№ of patients** | | **Effect** | | **Certainty** | **Importance** |
| --- | --- | --- | --- | --- | --- | --- | --- | --- | --- | --- | --- | --- |
| **№ of studies** | **Study design** | **Risk of bias** | **Inconsistency** | **Indirectness** | **Imprecision** | **Other considerations** | **EA** | **Control** | **Relative (95% CI)** | **Absolute (95% CI)** |  |  |
| **EA vs. control in severity of depression at the end of treatment** | | | | | | | | | | | | |
| 5 | randomised trials | not serious | serious^a^ | not serious | serious^b^ | none | 122 | 119 | - | MD **3.7**  (0.38 to 7.01) | ⨁⨁◯◯ Low | Important |

CI: confidence interval; MD: mean difference

a. Considerable heterogeneity (I^2^>75%)

b. Total event number less than 400

**Supplementary Table 5.** PRISMA 2020 Checklist

| **Section and Topic** | **Item #** | **Checklist item** | **Location where item is reported** |
| --- | --- | --- | --- |
| **TITLE** | | |  |
| Title | 1 | Identify the report as a systematic review. | P1 |
| **ABSTRACT** | | |  |
| Abstract | 2 | See the PRISMA 2020 for Abstracts checklist. | P1 |
| **INTRODUCTION** | | |  |
| Rationale | 3 | Describe the rationale for the review in the context of existing knowledge. | P2-3 |
| Objectives | 4 | Provide an explicit statement of the objective(s) or question(s) the review addresses. | P2-3 |
| **METHODS** | | |  |
| Eligibility criteria | 5 | Specify the inclusion and exclusion criteria for the review and how studies were grouped for the syntheses. | P3-4 |
| Information sources | 6 | Specify all databases, registers, websites, organisations, reference lists and other sources searched or consulted to identify studies. Specify the date when each source was last searched or consulted. | P3-4 |
| Search strategy | 7 | Present the full search strategies for all databases, registers and websites, including any filters and limits used. | P4-5, Supplementary Materials |
| Selection process | 8 | Specify the methods used to decide whether a study met the inclusion criteria of the review, including how many reviewers screened each record and each report retrieved, whether they worked independently, and if applicable, details of automation tools used in the process. | P5 |
| Data collection process | 9 | Specify the methods used to collect data from reports, including how many reviewers collected data from each report, whether they worked independently, any processes for obtaining or confirming data from study investigators, and if applicable, details of automation tools used in the process. | P5 |
| Data items | 10a | List and define all outcomes for which data were sought. Specify whether all results that were compatible with each outcome domain in each study were sought (e.g. for all measures, time points, analyses), and if not, the methods used to decide which results to collect. | P4 |
|  | 10b | List and define all other variables for which data were sought (e.g. participant and intervention characteristics, funding sources). Describe any assumptions made about any missing or unclear information. | P4 |
| Study risk of bias assessment | 11 | Specify the methods used to assess risk of bias in the included studies, including details of the tool(s) used, how many reviewers assessed each study and whether they worked independently, and if applicable, details of automation tools used in the process. | P5-6 |
| Effect measures | 12 | Specify for each outcome the effect measure(s) (e.g. risk ratio, mean difference) used in the synthesis or presentation of results. | P6 |
| Synthesis methods | 13a | Describe the processes used to decide which studies were eligible for each synthesis (e.g. tabulating the study intervention characteristics and comparing against the planned groups for each synthesis (item #5)). | P6 |
|  | 13b | Describe any methods required to prepare the data for presentation or synthesis, such as handling of missing summary statistics, or data conversions. | P6 |
|  | 13c | Describe any methods used to tabulate or visually display results of individual studies and syntheses. | P6 |
|  | 13d | Describe any methods used to synthesize results and provide a rationale for the choice(s). If meta-analysis was performed, describe the model(s), method(s) to identify the presence and extent of statistical heterogeneity, and software package(s) used. | P6-7 |
|  | 13e | Describe any methods used to explore possible causes of heterogeneity among study results (e.g. subgroup analysis, meta-regression). | P7 |
|  | 13f | Describe any sensitivity analyses conducted to assess robustness of the synthesized results. | P7 |
| Reporting bias assessment | 14 | Describe any methods used to assess risk of bias due to missing results in a synthesis (arising from reporting biases). | P6-7 |
| Certainty assessment | 15 | Describe any methods used to assess certainty (or confidence) in the body of evidence for an outcome. | N/A |
| **RESULTS** | | |  |
| Study selection | 16a | Describe the results of the search and selection process, from the number of records identified in the search to the number of studies included in the review, ideally using a flow diagram. | P7-8 |
|  | 16b | Cite studies that might appear to meet the inclusion criteria, but which were excluded, and explain why they were excluded. | N/A |
| Study characteristics | 17 | Cite each included study and present its characteristics. | P7-8, P21-23, Supplementary Materials |
| Risk of bias in studies | 18 | Present assessments of risk of bias for each included study. | P8 |
| Results of individual studies | 19 | For all outcomes, present, for each study: (a) summary statistics for each group (where appropriate) and (b) an effect estimate and its precision (e.g. confidence/credible interval), ideally using structured tables or plots. | P7-8 |
| Results of syntheses | 20a | For each synthesis, briefly summarise the characteristics and risk of bias among contributing studies. | P8-12 |
|  | 20b | Present results of all statistical syntheses conducted. If meta-analysis was done, present for each the summary estimate and its precision (e.g. confidence/credible interval) and measures of statistical heterogeneity. If comparing groups, describe the direction of the effect. | P8-12 |
|  | 20c | Present results of all investigations of possible causes of heterogeneity among study results. | P12 |
|  | 20d | Present results of all sensitivity analyses conducted to assess the robustness of the synthesized results. | P12-13 |
| Reporting biases | 21 | Present assessments of risk of bias due to missing results (arising from reporting biases) for each synthesis assessed. | P13 |
| Certainty of evidence | 22 | Present assessments of certainty (or confidence) in the body of evidence for each outcome assessed. | N/A |
| **DISCUSSION** | | |  |
| Discussion | 23a | Provide a general interpretation of the results in the context of other evidence. | P13-14 |
|  | 23b | Discuss any limitations of the evidence included in the review. | P14-15 |
|  | 23c | Discuss any limitations of the review processes used. | P14-15 |
|  | 23d | Discuss implications of the results for practice, policy, and future research. | P15 |
| **OTHER INFORMATION** | | |  |
| Registration and protocol | 24a | Provide registration information for the review, including register name and registration number, or state that the review was not registered. | P1 |
|  | 24b | Indicate where the review protocol can be accessed, or state that a protocol was not prepared. | N/A |
|  | 24c | Describe and explain any amendments to information provided at registration or in the protocol. | N/A |
| Support | 25 | Describe sources of financial or non-financial support for the review, and the role of the funders or sponsors in the review. | P16 |
| Competing interests | 26 | Declare any competing interests of review authors. | P15 |
| Availability of data, code and other materials | 27 | Report which of the following are publicly available and where they can be found: template data collection forms; data extracted from included studies; data used for all analyses; analytic code; any other materials used in the review. | P16 |

# 2.2
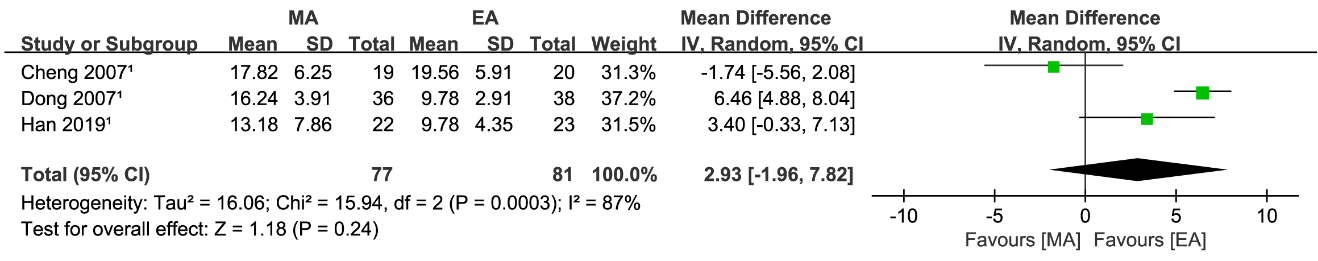
 Supplementary Figures


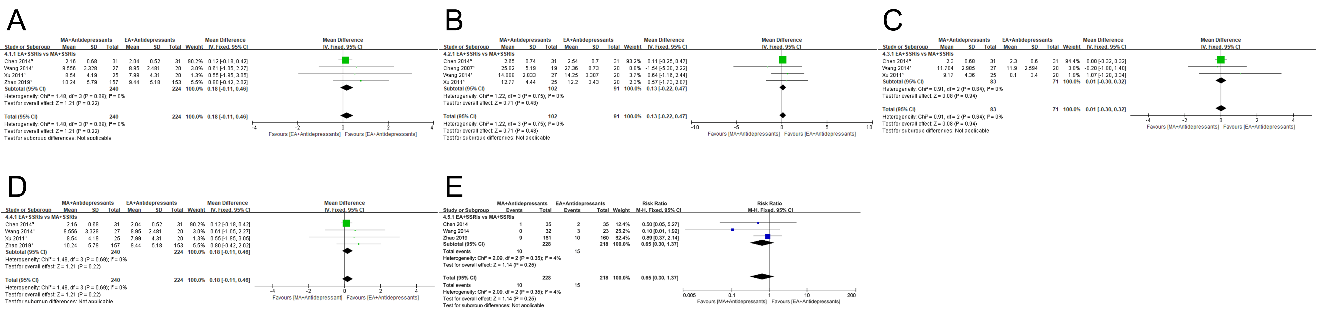
**Supplementary Figure 1.** Forest plot of comparison: EA vs. MA in severity of depression at the end of treatment. ¹ HAMD.

**Supplementary Figure 2.** Forest plot of comparison: EA+antidepressants vs. MA+antidepressants. (A) Severity of depression at the end of treatment. (B) Severity of depression at week 2 during treatment. (C) Severity of depression at week 4 during treatment. (D) Severity of depression at week 6 during treatment. (E) Adverse events. ¹ HAMD; ³ checklist 90 depression score.


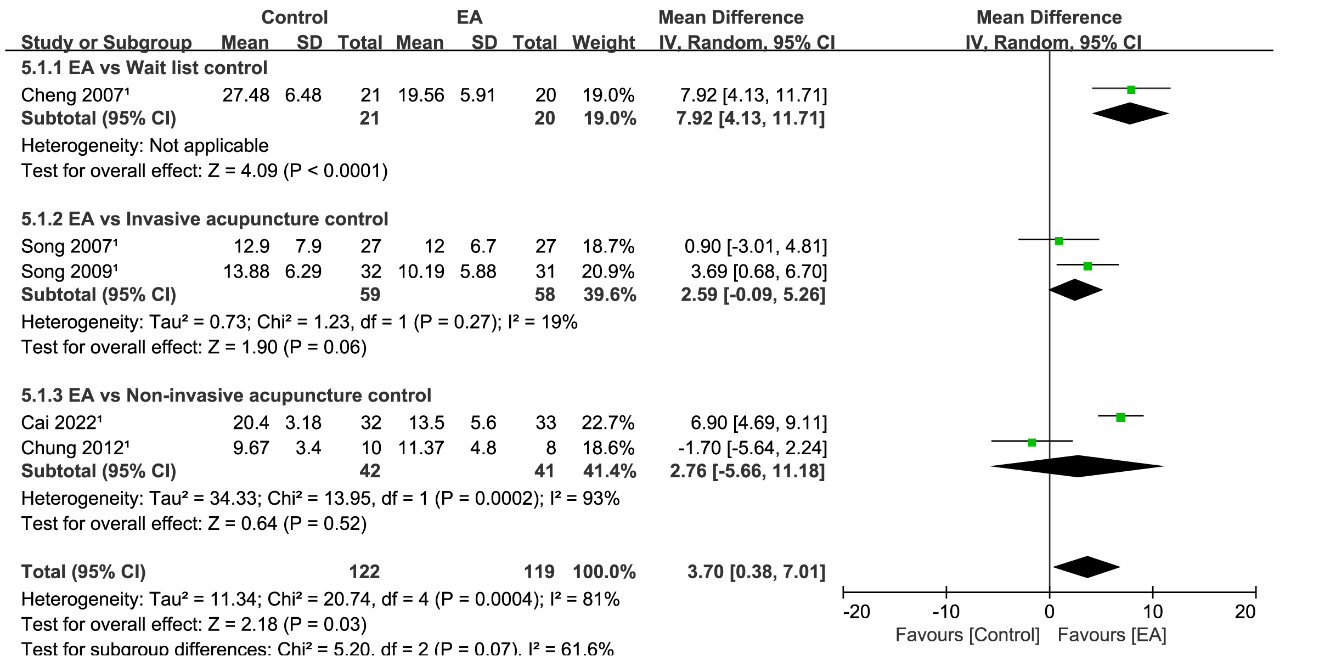


**Supplementary Figure 3.** Forest plot of comparison: EA vs. control in severity of depression at the end of treatment. ¹ HAMD.
